# Targeted therapy for capillary-venous malformations

**DOI:** 10.1038/s41392-024-01862-9

**Published:** 2024-06-17

**Authors:** Lola Zerbib, Sophia Ladraa, Antoine Fraissenon, Charles Bayard, Marina Firpion, Quitterie Venot, Sanela Protic, Clément Hoguin, Amandine Thomas, Sylvie Fraitag, Jean-Paul Duong, Sophie Kaltenbach, Estelle Balducci, Coline Lefevre, Patrick Villarese, Vahid Asnafi, Christine Broissand, Nicolas Goudin, Ivan Nemazanyy, Gwennhael Autret, Bertrand Tavitian, Christophe Legendre, Nadia Arzouk, Veronique Minard-Colin, Caroline Chopinet, Michael Dussiot, Denise M. Adams, Tristan Mirault, Laurent Guibaud, Paul Isenring, Guillaume Canaud

**Affiliations:** 1 Université Paris Cité, Paris, France; 2 INSERM U1151, Institut Necker-Enfants Malades, Paris, France; 3 Service d’Imagerie Pédiatrique, Hôpital Femme-Mère-Enfant, HCL, Bron, France; 4 CREATIS UMR 5220, Villeurbanne, 69100 France; 5 Service de Radiologie Mère-Enfant, Hôpital Nord, Saint Etienne, France; 6 Service d’Anatomie pathologique, Hôpital Necker-Enfants Malades, AP-HP, Paris, France; 7 Laboratoire d’Oncohématologie, Hôpital Necker-Enfants Malades, AP-HP, Paris, France; 8 Pharmacie, Hôpital Necker-Enfants Malades, AP-HP, Paris, France; 9 Necker Bio-Image Analysis, INSERM US24/CNRS UMS 3633, Paris, France; 10 Platform for Metabolic Analyses, Structure Fédérative de Recherche Necker, INSERM US24/CNRS UMS 3633, Paris, France; 11 Plateforme Imageries du Vivant, Université de Paris, PARCC, INSERM, Paris, France; 12 Service de Néphrologie, Transplantation Adultes, Hôpital Necker-Enfants Malades, AP-HP, Paris, France; 13 Service de Transplantation, Hôpital La Pitié Salpétrière, AP-HP, Paris, France; 14 Department of Pediatric and Adolescent Oncology, INSERM 1015, Gustave Roussy, Université Paris-Saclay, Villejuif, France; 15 Service de Physiologie & Explorations Fonctionnelles Cardiovasculaires, CHU de Lille, Lille, 59000 France; 16 INSERM U1163, Laboratory of Cellular and Molecular Mechanisms of Hematological Disorders and Therapeutic Implications, Laboratoire d’Excellence GR-Ex, Paris, France; 17 Division of Oncology, Comprehensive Vascular Anomalies Program, Children’s Hospital of Philadelphia, Philadelphia, PA USA; 18 Service de Médecine Vasculaire, hôpital Européen Georges-Pompidou, Paris, France; 19 Nephrology Research Group, L’Hôtel-Dieu de Québec Research Center, Department of Medicine, Faculty of Medicine, Laval University, Quebec, QC G1R2J6 Canada; 20 Unité de médecine translationnelle et thérapies ciblées, Hôpital Necker-Enfants Malades, AP-HP, Paris, France; 21 CNRS UMR8253, Institut Necker-Enfants Malades, Paris, France

**Keywords:** Translational research, Medical genetics

## Abstract

Sporadic venous malformations are genetic conditions primarily caused by somatic gain-of-function mutation of *PIK3CA* or *TEK*, an endothelial transmembrane receptor signaling through PIK3CA. Venous malformations are associated with pain, bleedings, thrombosis, pulmonary embolism, esthetic deformities and, in severe cases, life-threatening situations. No authorized medical treatment exists for patients with venous malformations. Here, we created a genetic mouse model of *PIK3CA*-related capillary venous malformations that replicates patient phenotypes. We showed that these malformations only partially signal through AKT proteins. We compared the efficacy of different drugs, including rapamycin, a mTORC1 inhibitor, miransertib, an AKT inhibitor and alpelisib, a PI3Kα inhibitor at improving the lesions seen in the mouse model. We demonstrated the effectiveness of alpelisib in preventing vascular malformations’ occurrence, improving the already established ones, and prolonging survival. Considering these findings, we were authorized to treat 25 patients with alpelisib, including 7 children displaying *PIK3CA* (*n* = 16) or *TEK* (*n* = 9)-related capillary venous malformations resistant to usual therapies including sirolimus, debulking surgical procedures or percutaneous sclerotherapies. We assessed the volume of vascular malformations using magnetic resonance imaging (MRI) for each patient. Alpelisib demonstrated improvement in all 25 patients. Vascular malformations previously considered intractable were reduced and clinical symptoms were attenuated. MRI showed a decrease of 33.4% and 27.8% in the median volume of PIK3CA and TEK malformations respectively, over 6 months on alpelisib. In conclusion, this study supports PI3Kα inhibition as a promising therapeutic strategy in patients with *PIK3CA* or *TEK*-related capillary venous malformations.

## Introduction

Venous malformations are a disabling condition characterized by low flow and slow growth vascular deformities that can be either localized or diffused. These malformations can be associated with hemorrhage, infections, thrombosis, pulmonary embolism, chronic pain, fatigue, functional impairments, disabilities and disseminated intravascular coagulation.^1^ Genetically, venous malformations are mainly due to gain-of-function mutations in either, *PIK3CA* or *Tie2R* (*TEK*).^1,2^ These mutations arise during embryonic development and result in somatic mosaicism.^3^. The estimated incidence of venous malformations is 1–5 in 10,000 births.^4,5^ Somatic mutations in *TEK* account for over 50% of sporadic unifocal venous malformations,^2,6^ while around 20% of cases are attributed to somatic mutations in *PIK3CA*.^7,8^

PI3Kα is a lipid kinase regulating signaling pathways involved in cell metabolism, motility, proliferation and survival.^9^ PI3Kα is predominantly activated via tyrosine kinase receptors. The PIK3CA gene encodes the 110-kDa catalytic alpha subunit of PI3Kα (p110α), responsible for converting phosphatidylinositol 4,5-bisphosphate (PtdIns(4,5)P2) into phosphatidylinositol 3,4,5-trisphosphate (PtdIns(3,4,5)P3; or PIP3) at the plasma membrane and subsequently recruits PDK1 through which AKT is phosphorylated on Thr^308^ residue. As for TEK, also called Tie2, it is a transmembrane receptor for angiopoietin-1 that signals through PI3Kα explaining why a gain-of-function mutation in this gene is associated with a phenotype that resembles the one seen with a gain-of-function mutation in *PIK3CA*.^10^

Venous malformations can be isolated or more complex when *PIK3CA* is the gene involved and are regrouped then under the appellation *PIK3CA*-related disorders.^11,12^ Histological examination of venous malformations usually shows thin-walled vessels that are variably dilated, commonly display thrombosis and occasionally contain phleboliths in the lumen and hemosiderin interpositions in the wall. Currently, treatments rely on compression, anticoagulation, sclerotherapy and surgery. Off-label use of rapamycin, an mTORC1 inhibitor, showed encouraging but variable results.^13,14^

Various mouse models are employed to understand the pathophysiology of capillary venous malformations. They include xenograft models with human endothelial cells bearing *TEK* or *PIK3CA* mutations.^7,15^ as well as mouse models created through a Cre/Lox approach.^8,16^ that recapitulate more or less faithfully the human disease.

A few years ago, a mouse model of PROS was successfully developed by our group using an inducible ubiquitously expressed *Cre* recombinase (*CAGG Cre*^*ER*^).^17^ The transgene used in this model was more potent than the mutations found in humans, enabling an accurate reproduction of the phenotype observed in PROS. With this mouse model, the effectiveness of alpelisib in the treatment of PROS was demonstrated in preclinical testing and subsequently in patients.^17–20^ These findings were further validated in the EPIK P1 clinical trial (NCT04285723), which led to the extension of alpelisib’s approval by the US FDA to patients with PROS aged two years and older.^21^ During the clinical trial, it was observed that the response to alpelisib varied among different tissues, with potentially significant efficacy seen in vascular tissues, particularly in veins.

Indeed, the recent progress in genetics and the emergence of targeted therapies hold promises for creating new opportunities for patients with venous malformations. Since the mouse models available to study venous malformations only partially reflect the human pathology, our aim here was to develop a novel preclinical model specifically mirroring the most severe manifestation of *PIK3CA*-related capillary venous malformations and that would enable us to closely examine the effects of alpelisib.

## Results

### A mouse model of PIK3CA gain of function in endothelial cells

We chose to develop a mouse model with a gain-of-function mutation in *PIK3CA* that specifically targets endothelial cells. For this purpose, we utilized the transgenic mouse strain R26StopFLP110* that is designed to express a dominant active *PIK3CA* transgene upon *Cre* recombination. By crossing these mice with *Tie2 Cre* mice, we thus generated *PIK3CA*^*Tie2-CreER*^ animals that carry a constitutively overactivated form of *PIK3CA* in capillaries and vein endothelial cells upon tamoxifen administration. To monitor Cre recombination, we further bred the *PIK3CA*^*Tie2-CreER*^ mice with *Gt(ROSA)26Sor*^*tm4(ACTB-tdTomato,-EGFP)Luo/J*^ mice in which an ubiquitously expressed cell membrane-localized tdTomato fluorescent protein is replaced by GFP following *Cre* recombination.^22^ For control purposes, littermate mice carrying only the *Tie2 Cre* recombinase without the *PIK3CA* transgene were used and will be referred to hereafter as *PIK3CA*^*WT*^ mice.

**Fig. 1:**
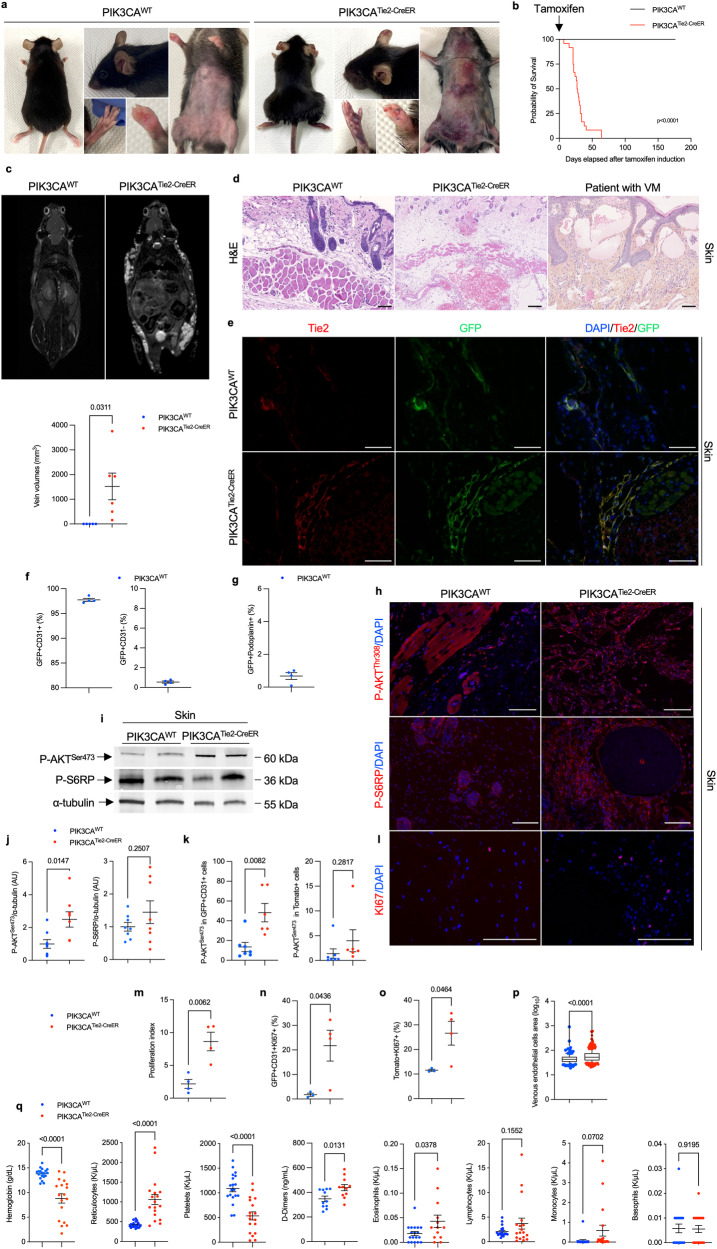
A mouse model of *PIK3CA*-related capillary venous malformations. **a** Representative photography of *PIK3CA*^*WT*^ and *PIK3CA*^*Tie2-CreER*^ mice 4 weeks after *Cre* recombination. **b** Kaplan–Meier survival curves of *PIK3CA*^*WT*^ and *PIK3CA*^*Tie2-CreER*^ mice (*n* = 25 per group). **c** Coronal whole-body T2-weighted fat saturated magnetic resonance images (MRI) of *PIK3CA*^*WT*^ and *PIK3CA*^*Tie2-CreER*^ mice (*n* = 4–6 mice per group) 5 weeks after *Cre* recombination. Volumetric quantification of the vascular malformations. **d** Representative hematoxylin and eosin (H&E) staining of the skin of *PIK3CA*^*WT*^ and *PIK3CA*^*Tie2-CreER*^ mice and patient with venous malformation (VM). Large irregular and dilated vessels filled with red blood cells were visible in the skin of *PIK3CA*^*Tie2-CreER*^ mice. Scale bar: 40 μm. **e** Representative coimmunofluorescence staining of Tie2 and GFP in the skin of *PIK3CA*^*WT*^ and *PIK3CA*^*Tie2-CreER*^ mice. Scale bar: 10 μm. **f** GFP is express in CD31+ cells as assessed by flow cytometry experiments of cells isolated from the skin of *PIK3CA*^*WT*^ (*n* = 4 mice). **g** GFP and podoplanin staining using flow cytometry experiments of cells isolated from the skin of *PIK3CA*^*WT*^ (*n* = 4 mice). **h** Representative immunofluorescence staining of P-AKT^Thr308^ and P-S6RP in the skin of *PIK3CA*^*WT*^ and *PIK3CA*^*Tie2-CreER*^ mice. Scale bar: 10 μm. **i** Western blot and **j** quantification of P-AKT^Ser473^ and P-S6RP in the skin of *PIK3CA*^*WT*^ and *PIK3CA*^*Tie2-CreER*^ mice (*n* = 5–8 mice per group). **k** Flow cytometry experiments showing the percentage of GFP + CD31+ cells isolated from the skin of *PIK3CA*^*WT*^ and *PIK3CA*^*Tie2-CreER*^ mice expressing P-AKT^Ser473^ (*n* = 6–7 mice per group). **l** Representative immunofluorescence staining of KI67 in the skin of *PIK3CA*^*WT*^ and *PIK3CA*^*Tie2-CreER*^ mice. Scale bar: 10 μm and **m** quantification (*n* = 4 mice per group). **n** Flow cytometry experiments showing the percentage of GFP + CD31+ cells isolated from the skin of *PIK3CA*^*WT*^ and *PIK3CA*^*Tie2-CreER*^ mice expressing KI67 (*n* = 3–4 mice per group). **o** Flow cytometry experiments showing the percentage of Tomato+ cells isolated from the skin of *PIK3CA*^*WT*^ and *PIK3CA*^*Tie2-CreER*^ mice expressing KI67 (*n* = 3–4 mice per group). **p** Quantification of GFP positive cell surface isolated from *PIK3CA*^*WT*^ and *PIK3CA*^*Tie2-CreER*^ mice (*n* = 5 mice per group). **q** Complete blood count and D-Dimers measurement in *PIK3CA*^*WT*^ and *PIK3CA*^*Tie2-CreER*^ mice (*n* = 11 per group)

To address developmental concerns, we utilized four-week-old mice and administered a daily dose of 40 mg.kg-1 tamoxifen for five consecutive days to induce *Cre* recombination. After observing a delay of 2 to 3 weeks following Cre recombination, we noticed progressive and visible generalized subcutaneous capillary and venous malformations in *PIK3CA*^*Tie2-CreER*^ mice (Fig. 1a), accompanied by excessive weight gain (Supplementary Fig. 1a). Both male and female mice exhibited these characteristics (Supplementary Fig. 1a). The malformations showed rapid growth and were associated with a significant reduction in survival compared to controls (Fig. 1b). To further investigate the extent of the malformations, we conducted whole-body T2-weighted magnetic resonance imaging (MRI) at 5 weeks after *Cre* induction. These studies revealed the presence of disseminated superficial capillaries and deep venous malformations (Fig. 1c and Supplementary Fig. 1b). Necropsy examinations demonstrated subcutaneous hemorrhage all linked to disseminated vascular malformations in the peri-laryngeal space, lower and/or upper limbs, and urogenital area (Supplementary Fig. 1c).

Histological analysis of the capillary and venous malformations revealed dilated and irregular vessels with disorganized tissue structure, endothelial hyperplasia, blood-filled lumens, hemosiderin interposition, and features of hemophagocytosis (Supplementary Fig. 1d). In some mice, local clotting was accompanied by pulmonary embolism (Supplementary Fig. 1e, f). In the malformations, we also noticed varying degrees of adipose tissue overgrowth, lymphatic compounds, and αSMA positive cells (Supplementary Fig. 1f). These abnormalities closely resemble the ones that are seen in patients with capillary venous malformations (Supplementary Fig. 1g).

We then verified *Cre* recombination through GFP and p110* expression. Coimmunostaining (Fig. 1E and Supplementary Fig. 2a), Western blot (Supplementary Fig. 2b) and flow cytometry experiments confirmed that the *Cre* recombinase was specifically expressed in CD31 positive cells (97.8 ± 0.31% mean ± SEM, *n* = 4 mice). Conversely 0.54 ± 0.1% of the cells were GFP + CD31− showing indeed the endothelial cell expression of the Cre recombinase (*n* = 4 mice) (Fig. 1f). Importantly, no Cre expression was detected in lymphatic endothelial cells using flow cytometry experiments (0.67 ± 0.2%, mean ± SEM, *n* = 4 mice) (Fig. 1g). Indeed, the presence of adipose tissue overgrowth and lymphatic anomalies suggests potential communication between endothelial cells and surrounding tissues or cell recruitment. In this regard, we noticed infiltration of immune cells within the lesions as indicated by the presence of CD3+ cells (Supplementary Fig. 2c) and F4/80-expressing cells (that is, macrophages). Macrophages were visible either within the lumen or in the close vicinity of dilated vessels (Supplementary Fig. 2d–f), with a significant proportion of CD163, a receptor involved in clearing and endocytosis of hemoglobin/haptoglobin complexes (Supplementary Fig. 2d–f). Flow cytometry experiments showed that the Cre recombinase was not expressed in CD45+ (0.21±0.03% mean ± SEM, *n* = 4 mice) or CD34+(0.09±0.01%, mean±SEM, *n* = 4 mice) bone marrow cells (Supplementary Fig. 2g, h).

Coimmunofluorescence experiments using GFP, COUP-TFII, CD31, Podoplanin, Ephrin B2 and Endomucin on 30 μm sections revealed severe disorganization in the 3D structure of the skin vessels (Supplementary Figs. 3 and 4).

Immunofluorescence (Fig. 1h), Western blot (Fig. 1i, j) and flow cytometry studies (Fig. 1k) revealed activation of the AKT and mTOR pathways in the endothelial cells of *PIK3CA*^*Tie2-CreER*^ mice by contrast to *PIK3CA*^*WT*^. Mechanistically, PI3Kα is known to activate cell growth and proliferation. We investigated cell proliferation using KI67 staining (Fig. 1l, m, Supplementary Fig. 5a, b) and flow cytometry experiments. In control mice, we noted that 1.7 ± 0.6% (mean ± SEM, *n* = 4) of CD31 + GFP+ and 11.5 ± 0.4% (mean ± SEM, *n* = 4) of the Tomato+ cells co-expressed KI67 (Fig. 1n, o). Remarkably, in PIK3CA^Tie2-CreER^ mice we observed an elevated proliferation rate in the CD31 + GFP+ population (21.7 ± 6.2% were positive for KI67) as well as in Tomato+ cells (26.6±4.8% were positive for KI67) (Fig. 1n, o). This finding indicates the proliferation of endothelial cells and supports again a potential crosstalk between mutated and surrounding wild-type cells. Using Amnis ImageStream® system, we also found that mutated endothelial cells isolated from *PIK3CA*^*Tie2-CreER*^ mice were hypertrophic by contrast to controls (Fig. 1p and Supplementary Fig. 5c).

Finally, complete blood counts in *PIK3CA*^*Tie2-CreER*^ mice revealed significant regenerative anemia, a low platelet count, and elevated D-Dimers levels, consistent with the possibility of disseminated intravascular coagulation, as well as slight hypereosinophilia. All of these abnormalities are also consistent with what is observed in patients with venous malformations (Fig. 1q).^23,24^

We thus have successfully established a mouse model that accurately replicates the phenotype of patients who are affected by somatic or inherited forms of capillary venous malformations. This model should offer the advantage of providing mechanistic insight into both the *PIK3CA*- and *TEK*-related types of vascular diseases.

We then investigated whether the *PIK3CA* gain-of-function mutation in capillaries and veins would lead to detectable changes in circulating metabolites that could potentially serve as biomarkers for disease progression. For this purpose, we collected plasma samples from both *PIK3CA*^*WT*^ and *PIK3CA*^*Tie2-CreER*^ mice after a 12-hour fast and subjected them to thorough metabolomic analysis. *PIK3CA*^*Tie2-CreER*^ mice were found to exhibit activated anabolic pathways, resulting in the accumulation of various amino acids, including arginine, histidine, phenylalanine, thymidine, threonine, 5-adenosyl-homocysteine, valine, orotic acid, acetyl-lysine, methyl-lysine, lysine, and oxoadipate, along with the metabolic intermediates of these amino acids. Additionally, there was evidence of increased mitochondrial respiration in these mice, as indicated by the plasma accumulation of succinic acid and acetyl-carnitine, and activation of the urea cycle as indicated by higher circulating levels of arginine and urate compared to controls (Supplementary Fig. 6). As such, circulating metabolites could serve as potential biomarkers for monitoring disease progression in *PIK3CA-*capillary venous malformations.

In humans, a great number of *PIK3CA* variants have been identified and found to exert varying effects on the recruitment of the AKT pathway.^25^ To simulate a higher degree of AKT recruitment, we also created *PIK3CA*^*Tie2-HO*^ mice that bear homozygous *PIK3CA* mutations in capillaries and venous endothelial cells. After *Cre* recombination, a more severe phenotype emerged, characterized by the rapid occurrence of disseminated and voluminous capillary venous malformations with reduced life expectancy (Supplementary Fig. 7a–c). Whole-body T2-weighted MRI conducted five weeks after induction further confirmed the presence of extensive disseminated venous malformations (Supplementary Fig. 7d). Histological examination revealed severe widespread clotting and pulmonary embolism (Supplementary Figs. 7e and 4f), phosphorylation assays of tissues, robust activation of the AKT pathway (Supplementary Fig. 7g, h) and complete blood count, profound regenerative anemia (Supplementary Fig. 7i).

### PIK3CA signals partially through AKT1 and not AKT2 in venous endothelial cells

In mammals, AKT is encoded by three distinct genes: *AKT1*, *AKT2*, and *AKT3*, with *AKT1* being the main isoform in endothelial cells. To explore whether *PIK3CA* gain-of-function mutations primarily signal via AKT or other downstream targets, we conducted a series of experiments using genetically modified mice.

**Fig. 2:**
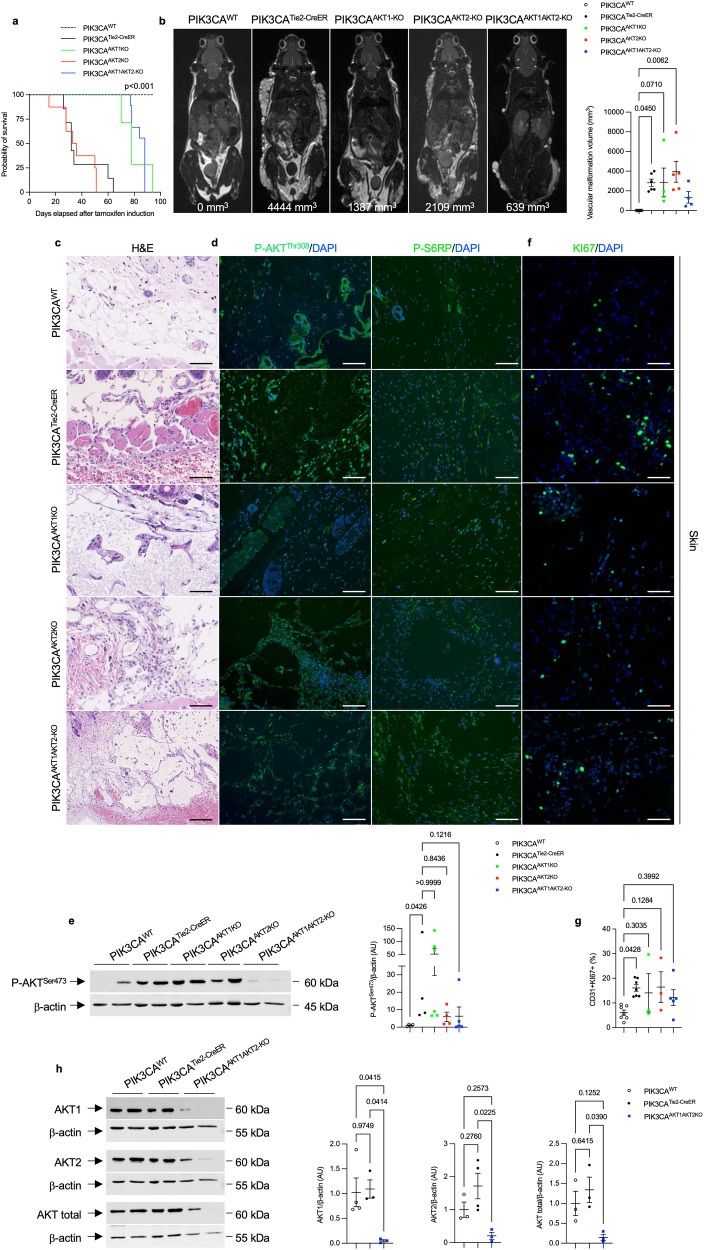
*PIK3CA*-related capillary venous malformations in *PIK3CA*^*Tie2-CreER*^ mice only partially signal through AKT proteins. **a** Kaplan–Meier survival curves of *PIK3CA*^*WT*^, *PIK3CA*^*Tie2-CreER*^, *PIK3CA*^*AKT1KO*^, *PIK3CA*^*AKT2KO*^ and *PIK3CA*^*AKT1AKT2-KO*^ mice (*n* = 20 per group). **b** Coronal whole-body T2 weighted magnetic resonance images (MRI) of *PIK3CA*^*WT*^, *PIK3CA*^*Tie2-CreER*^, *PIK3CA*^*AKT1KO*^, *PIK3CA*^*AKT2KO*^ and *PIK3CA*^*AKT1AKT2-KO*^ mice 6 weeks after *Cre* recombination. Volumetric quantification of the vascular malformations (*n* = 4–6 mice per group). **c** Representative hematoxylin and eosin (H&E) staining of the skin of *PIK3CA*^*WT*^, *PIK3CA*^*Tie2-CreER*^, *PIK3CA*^*AKT1KO*^, *PIK3CA*^*AKT2KO*^ and *PIK3CA*^*AKT1AKT2-KO*^ mice. Scale bar: 10 μm. **d** Representative P-AKT^Ser473^ in the skin of *PIK3CA*^*WT*^, *PIK3CA*^*Tie2-CreER*^, *PIK3CA*^*AKT1KO*^, *PIK3CA*^*AKT2KO*^ and *PIK3CA*^*AKT1AKT2-KO*^ mice. Scale bar: 10μm.*: Vascular malformation. **e** Western blot and quantification of P-AKT^Ser473^ and P-S6RP in the skin of *PIK3CA*^*WT*^, *PIK3CA*^*Tie2-CreER*^, *PIK3CA*^*AKT1KO*^, *PIK3CA*^*AKT2KO*^ and *PIK3CA*^*AKT1AKT2-KO*^ mice (*n* = 4–5 mice per group). **f** Representative immunofluorescence staining of KI67 in the skin of *PIK3CA*^*WT*^, *PIK3CA*^*Tie2-CreER*^, *PIK3CA*^*AKT1KO*^, *PIK3CA*^*AKT2KO*^ and *PIK3CA*^*AKT1AKT2-KO*^ mice. Scale bar: 10 μm. **g** Flow cytometry experiments showing the percentage of GFP + CD31+ cells isolated from the skin of the different mouse models expressing KI67 (*n* = 3–4 mice per group). (*n* = 3–7 mice per group). **h** Western blot and quantification of AKT1 and AKT2 in vessels of *PIK3CA*^*WT*^, *PIK3CA*^*Tie2-CreER*^ and *PIK3CA*^*AKT1AKT2-KO*^ mice (*n* = 3–4 mice per group)

First, we interbred *AKT1*^*flox/flox*^ with *PIK3CA*^*Tie2R-CreER*^ mice to create a *PIK3CA*^*Tie2R-CreER-AKT1KO*^ model (referred to as *PIK3CA*^*AKT1KO*^). Upon inducing *Cre* induction, we observed that *AKT1* deletion in capillaries and veins led to a moderate extension of lifespan (median survival of 80 days) and only delayed the occurrence of vascular malformations (Fig. 2a and Supplementary Fig. 8a) as MRI performed at 6 weeks still revealed diffused venous malformations (Fig. 2b and Supplementary Fig. 8b). Histologically, the malformations were similar to the ones observed in *PIK3CA*^*Tie2R-CreER*^ mice (Fig. 2c). AKT pathway recruitment exhibited no reduction (Fig. 2d, e), and cell proliferation rates remained unaffected by deletion of *AKT1* (Fig. 2f, g). *AKT1* deletion did not rescue complete blood count anomalies either (Supplementary Fig. 8c). These findings suggest that the AKT1 isoform only plays a partial role in the *PIK3CA*-driven phenotype of vascular malformations.

Next, we investigated the role of the AKT2 isoform by interbreeding *AKT2*^*−/−*^ with *PIK3CA*^*Tie2R-CreER*^ mice to obtain *PIK3CA*^*Tie2R-CreER-AKT2KO*^ model (referred to as *PIK3CA*^*AKT2KO*^). Following the induction of *Cre*, we observed that *PIK3CA*^*AKT2KO*^ mice exhibited the same clinical, biological, radiological and histological manifestations as *PIK3CA*^*Tie2R-CreER*^ mice (Fig. 2a–c and Supplementary Fig. 8a, c). AKT phosphorylation in tissues and proliferation rates were also modestly affected in *PIK3CA*^*Tie2R-CreER*^ animals (Fig. 2d, e). Thus, *AKT2* deletion did not impact the *PIK3CA*-vascular malformation phenotype.

Finally, we investigated the simultaneous deletion of both *AKT1* and *AKT2* isoforms by interbreeding *PIK3CA*^*AKT1KO*^ with *PIK3CA*^*AKT2KO*^ mice. The resulting *PIK3CA*^*AKT1AKT2-KO*^ pups were born in the expected Mendelian ratio and did not display any particular phenotype during the first few weeks of life. At 4 weeks of age, we activated *Cre* recombinase, and over time, *PIK3CA*^*AKT1AKT2-KO*^ mice began to develop capillary and venous malformations (Supplementary Fig. 8a). Whole-body MRI performed 6 weeks after *Cre* recombination revealed diffuse malformations (Fig. 2b and Supplementary Fig. 8b). Similar to *PIK3CA*^*AKT1KO*^ mice, *AKT1* and *AKT2* deletion in capillaries and veins was associated with a moderate extension of lifespan (Fig. 2z). Histology and blood examination confirmed severe venous malformation with intravascular coagulation (Fig. 2c and Supplementary Fig. 8c). Once again, a modest reduction in AKT phosphorylation but not in the proliferation rate were observed (Fig. 2d–g). Western blot analysis showed that while *AKT1* and *AKT2* were successfully deleted in endothelial cells (Fig. 2h), AKT protein was still expressed, indicating possible compensation by *AKT3*. Indeed, we concluded that, genetic ablation of both isoforms AKT1 and 2 was associated with delayed occurrence of the malformations.

### Targeted therapy for venous malformations in PIK3CA related venous malformations

We proceeded to investigate whether targeted therapies.^16^ could improve the outcome of *PIK3CA*^*Tie2R-CreER*^ mice. For this purpose, we compared the effects of three pharmacological inhibitors: rapamycin, miransertib, and alpelisib, targeting mTORC1,^26^ pan AKT, and PI3Kα, respectively. Four weeks after tamoxifen administration, mice were randomly assigned to one of the treatment groups.

**Fig. 3:**
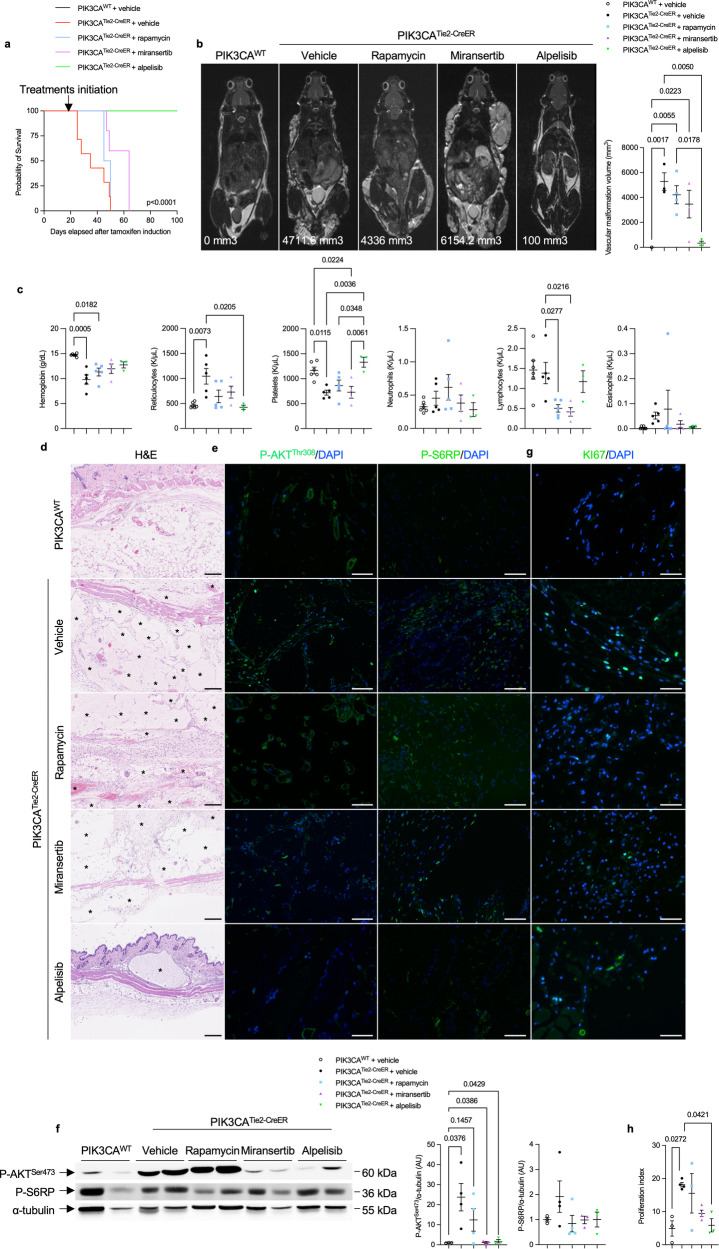
Targeted therapies for capillary venous malformations in *PIK3CA*^*Tie2-CreER*^ mice. **a** Kaplan–Meier survival curves of *PIK3CA*^*WT*^ and *PIK3CA*^*Tie2-CreER*^ mice treated with either vehicle, rapamycin, miransertib or alpelisib (*n* = 12 per group). **b** Coronal whole-body T2-weighted magnetic resonance images (MRI) of *PIK3CA*^*WT*^ and *PIK3CA*^*Tie2-CreER*^ mice 6 weeks after *Cre* recombination treated with either vehicle, rapamycin, miransertib or alpelisib. Volumetric quantification of the vascular malformations (*n* = 3-4 mice per group). **c** Complete blood count in *PIK3CA*^*WT*^ and *PIK3CA*^*Tie2-CreER*^ mice treated with either vehicle, rapamycin, miransertib or alpelisib (*n* = 3–5 mice per group). **d** Representative hematoxylin and eosin (H&E) staining of the skin of *PIK3CA*^*WT*^ and *PIK3CA*^*Tie2-CreER*^ mice treated with either vehicle, rapamycin, miransertib or alpelisib. Scale bar: 10 μm. **e** Representative P-AKT^Thr308^ and P-S6RP immunostaining in the skin of *PIK3CA*^*WT*^and *PIK3CA*^*Tie2-CreER*^ mice treated with either vehicle, rapamycin, miransertib or alpelisib. Scale bar: 10 μm. **f** Western blot and quantification of P-AKT^Ser473^ and P-S6RP of the skin of *PIK3CA*^*WT*^and *PIK3CA*^*Tie2-CreER*^ mice treated with either vehicle, rapamycin, miransertib or alpelisib (*n* = 3–4 per group). **g** Representative immunofluorescence staining of KI67 in the skin of *PIK3CA*^*WT*^and *PIK3CA*^*Tie2-CreER*^ mice treated with either vehicle, rapamycin, miransertib or alpelisib. Scale bar: 10 μm. **h** Proliferation index quantification (*n* = 3–4 mice per group)

Rapamycin, which is used off-label to treat various forms of venous malformations in patients, did not show any impact on the volume and histological aspects of venous malformations, on intravascular coagulation, or on overall survival when administered daily (Fig. 3a–d, Supplementary Fig. 9a, b). Rapamycin led to a reduction in S6RP phosphorylation in tissues, but AKT phosphorylation (Fig. 3e, f) and proliferation rate (Fig. 3g, h) remained very high.

Miransertib, which had shown encouraging preclinical results,^16^ resulted in a slight reduction in venous malformation volumes (Fig. 3b, Supplementary Figs. 9a, b) and modest lifespan extension (Fig. 3a). However, it did not significantly impact platelet level, anemia or the histological abnormalities (Fig. 3c, d). Western blot and immunofluorescence studies did reveal a reduction in AKT and S6RP phosphorylation (Fig. 3e, f) with a modest impact on proliferation (Fig. 3g, h). These findings suggest again that *PIK3CA*-capillary venous malformations are not solely dependent on AKT for their development

In contrast to these 2 previous drugs, alpelisib showed significant efficacy. It led to prolonged survival (Fig. 3a), a notable reduction in venous malformations volume as confirmed by MRI (Fig. 3b, Supplementary Fig. 9a, b) and histological analyses (Fig. 3d) and to the reversal of intravascular coagulation biologically (Fig. 3c). In the affected tissues, the AKT pathway and cell proliferation were completely inhibited (Fig. 3e–h). These results demonstrate that alpelisib outperformed rapamycin and miransertib in treating *PIK3CA*^*Tie2R-CreER*^ mice, showing promising potential as a therapeutic option for the management of vascular malformations in particular.

### Alpelisib prevents venous malformations in PIK3CA mouse model

**Fig. 4:**
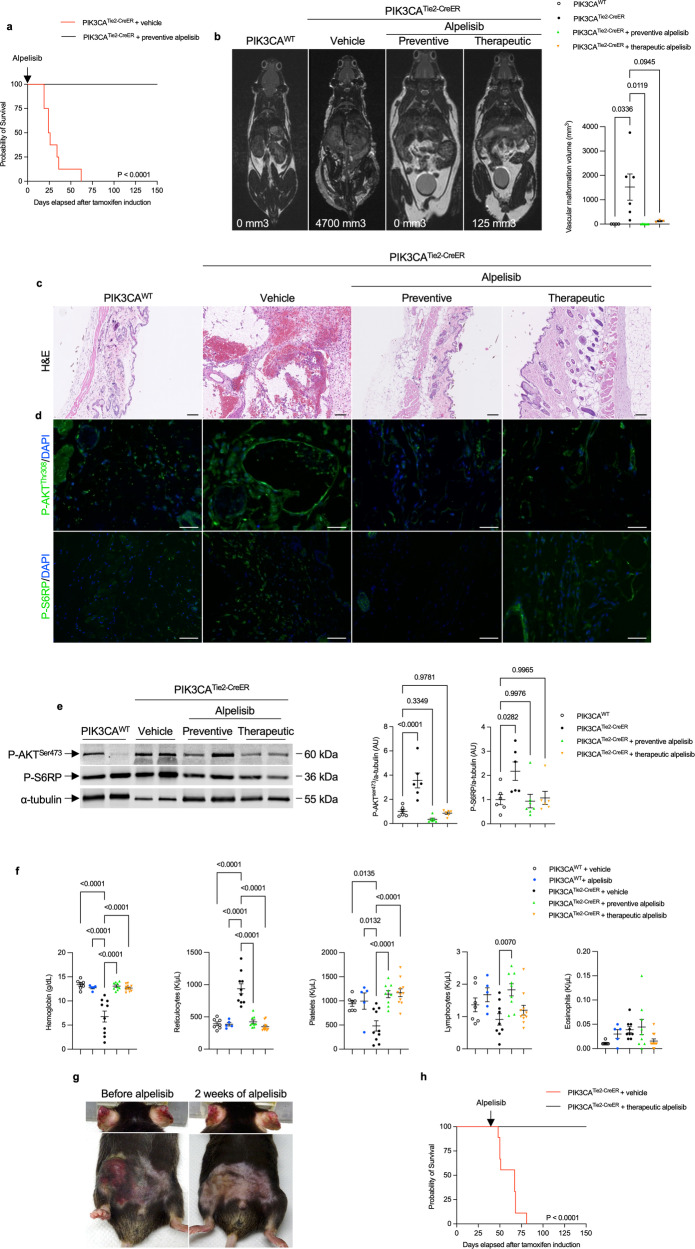
Alpelisib improves and prevent capillary venous malformations in *PIK3CA*^*Tie2-CreER*^ mice. **a** Kaplan–Meier survival curves of *PIK3CA*^*WT*^ and *PIK3CA*^*Tie2-CreER*^ mice treated with either vehicle or preventive alpelisib (*n* = 12 per group). **b** Coronal whole-body T2-weighted magnetic resonance images (MRI) of *PIK3CA*^*WT*^ and *PIK3CA*^*Tie2-CreER*^ mice 6 weeks after *Cre* recombination treated with either vehicle, preventive or therapeutic alpelisib (*n* = 4–6 mice per group). Volumetric quantification of the vascular malformations. **c** Representative hematoxylin and eosin (H&E) staining of the skin of *PIK3CA*^*WT*^ and *PIK3CA*^*Tie2-CreER*^ mice treated with either vehicle, preventive or therapeutic alpelisib. Scale bar: 10 μm. **d** Representative P-AKT^Thr308^ and P-S6RP immunostaining in the skin of *PIK3CA*^*WT*^and *PIK3CA*^*Tie2-CreER*^ mice treated with either vehicle, preventive or therapeutic alpelisib. Scale bar: 10 μm. **e** Western blot and quantification of P-AKT^Ser473^ and P-S6RP in skin of *PIK3CA*^*WT*^and *PIK3CA*^*Tie2-CreER*^ mice treated with either vehicle, preventive or therapeutic alpelisib (*n* = 6–7 mice per group). **f** Complete blood count of *PIK3CA*^*WT*^and *PIK3CA*^*Tie2-CreER*^ mice treated with either vehicle, preventive or therapeutic alpelisib (*n* = 5–10 mice). **g** Representative photography of *PIK3CA*^*Tie2-CreER*^ mice before and two weeks after alpelisib initiation. **h** Kaplan–Meier survival curves of *PIK3CA*^*WT*^ and *PIK3CA*^*Tie2-CreER*^ mice treated with either vehicle or therapeutic alpelisib (*n* = 12 per group)

We next aimed to assess whether alpelisib could be effective in both preventing and improving vascular malformations in *PIK3CA*^*Tie2-CreER*^ mice. To conduct a preventive study, we began to administer alpelisib vs. a vehicle 48 h after *Cre* induction and maintained it for 12 weeks. Alpelisib-treated *PIK3CA*^*Tie2-CreER*^ mice displayed a visibly normal appearance and weight gain profile with extended lifespan compared to vehicle-treated *PIK3CA*^*Tie2-CreER*^ mice (Fig. 4a, Supplementary Fig. 10a, b). MRI conducted after 6 weeks of alpelisib demonstrated the absence of vascular malformations (Fig. 4b, Supplementary Fig. 10c, d) and similar conclusions could be drawn based on necropsy examination (Fig. 4c). Alpelisib also effectively inhibited AKT and mTOR (Fig. 4d, e and Supplementary Fig. 10e), improved vascular anomalies (Supplementary Fig. 11a) and prevented blood cell count abnormalities from occurring (Fig. 4f).

For the therapeutic study, alpelisib was administered to *PIK3CA*^*Tie2-CreER*^ mice 4 weeks after Cre induction when vascular malformations were already visible. This treatment led to a rapid decrease in body weight in alpelisib-treated *PIK3CA*^*Tie2-CreER*^ mice (Supplementary Fig. 10a), clinical improvement of the vascular malformations (Fig. 4g and Supplementary Fig. 10b), and extended lifespan (Fig. 4h). MRI performed 6 weeks after the start of alpelisib treatment showed a significant decrease in the volume of vascular malformations compared to vehicle-treated mice (Fig. 4b, Supplementary Fig. 10c, d). At the time of sacrifice, alpelisib-treated *PIK3CA*^*Tie2-CreER*^ mice exhibited small vascular dilation (Fig. 4c), and AKT and S6RP phosphorylation were reduced (Fig. 4d, e and Supplementary Fig. 10e). Similar to the preventive study, blood anomalies and vessel malformations were corrected (Fig. 4f and Supplementary Fig. 11a). Furthermore, we investigated the impact of alpelisib on the plasma metabolites identified as dysregulated in *PIK3CA*^*Tie2-CreER*^ mice. These metabolites completely or partially normalized under alpelisib in both the preventive and therapeutic conditions (Supplementary Figs. 12 and 13), indicating that they hold potential as early biomarkers of disease progression or regression in the mouse model. Alpelisib had no particular impact on the control mice. Finally, to explore whether vascular malformations could recur following the interruption of alpelisib, we induced Cre recombination in 4 male mice, treated them with alpelisib at 8 weeks post tamoxifen induction when they were severely affected for a period of 16 weeks, and then stopped the drug. In the following weeks, the mice restarted to gain body weight with the development of diffuse vascular malformations (Supplementary Fig. 11b). Indeed, alpelisib halted disease development but did not correct the genetic anomaly.

### Alpelisib improves capillary venous malformations in patients with PIK3CA or TEK variants

**Fig. 5:**
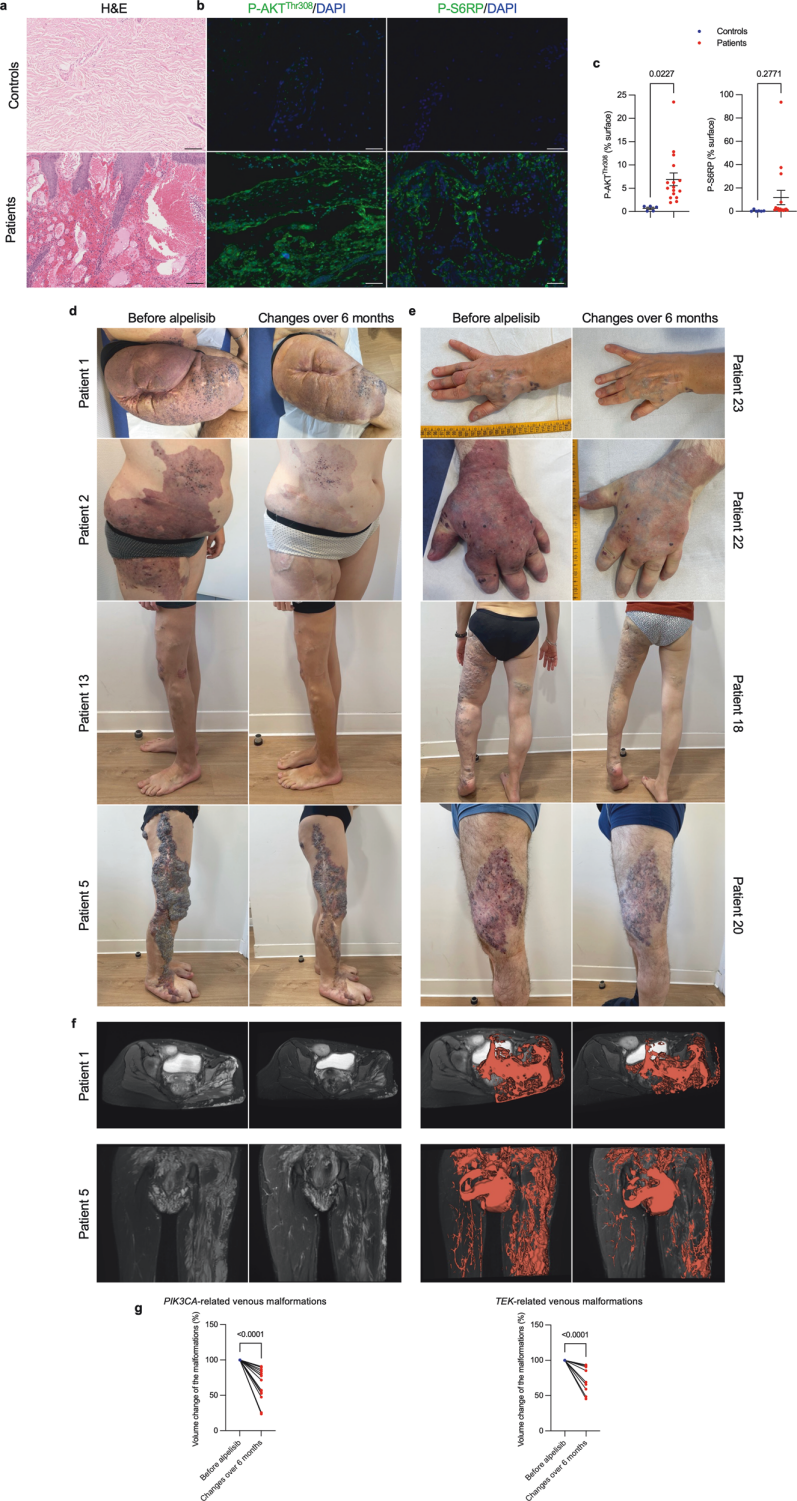
Alpelisib improves patients with *PIK3CA* or *TEK*-related capillary venous malformations. **a** Representative Hematoxylin and eosin (H&E) staining and **b** Immunofluorescence of P-AKT^Thr308^ and P-S6RP in skin biopsies performed in controls and in patients with *PIK3CA*-related capillary venous malformation. Scale bar: 10 μm. **c** Immunofluorescence quantification (*n* = 6 controls and 6 patients). AU Arbitrary units. **d** Representative photographs of the morphological changes observed in patients with *PIK3CA*-related capillary venous malformations receiving alpelisib for 6 months. **e** Representative photographs of the morphological changes observed in patients with *TEK*-related capillary venous malformations receiving alpelisib for 6 months. **f** Transversal (upper panel) and coronal (lower panel) T2-weighted fat saturated MRI sequence of patient 1 and 5 before and after alpelisib introduction. In red, segmentation in 2D (left panel) and 3D (right panel). **g** Percentage change of the volume of the preselected lesion in patients with *PIK3CA*-related capillary venous malformations (left panel) and *TEK*-related capillary venous malformations (right panel)

After obtaining encouraging results, we received authorization from the French regulatory agency (ANSM) and The Comité de convenance du CHU de Québec to treat a total of 25 patients, both pediatric and adult, who had venous malformations associated with either *PIK3CA* mutation (*n* = 16) or *Tie2R* (*TEK*) mutation (*n* = 9), all demonstrating AKT pathway recruitment within the capillary venous malformations (Fig. 5a–c). However, we observed no difference in KI67 staining between controls and patients (Supplementary Fig. 14). The clinical and demographical characteristics of these patients are summarized in Tables 1 and 2, but briefly there were 10 women, 7 pediatric patients and the mean age was 29.12 years old (SD ± 15.49). All patients were previously heavily treated with multiple surgical, radiological procedures and treatment based on rapamycin for a minimum period of time of 6 months (Tables 1 and 2). Patients were selected because of rapamycin inefficiency (disease progression or uncontrolled symptoms or serious adverse events). All patients underwent a Doppler ultra sound and an MRI before initiating treatment with alpelisib and then a second MRI approximately 6 months after starting the medication. The dosage of alpelisib administered ranged from 50 to 250 mg, adjusted according to age based on the EPIK P1 clinical trial.^21^ After the introduction of alpelisib, all patients experienced improvements in symptoms such as pain or bleedings. Furthermore, there was a noticeable discoloration and reduction in the volume of vascular malformations observed in the patients (Tables 1 and 2, Fig. 5d, e and Supplementary Fig. 15). Specifically, *PIK3CA*-related venous malformations showed a mean reduction of 33.4% (SD, ± 22.1) from the baseline at 6 months, while *TEK-*related venous malformations demonstrated a mean reduction of 27.8% (SD, ±18.9) (Tables 1 and 2, Fig. 5f and Supplementary Fig. 16). Finally, we explored the metabolomic changes in patients following alpelisib introduction. Notably, we identified changes in multiple metabolites that aligned with the ones observed in the mouse model such as aspartate, cystine, glycerol, guanosine, inosine or succinic acid (Supplementary Fig. 17). These metabolic compounds could potentially serve as the foundation for disease improvement biomarkers.

**Table 1:** Patient characteristics with *PIK3CA*-related venous malformations

	Patient 1	Patient 2	Patient 3	Patient 4	Patient 5	Patient 6	Patient 7	Patient 8	Patient 9	Patient 10	Patient 11	Patient 12
Age (years)	34	24	29	3	16	49	43	23	42	43	15	44
DNA changes	c.1624G>A	c.1633G>A	c.1638G>T	c.1633G>A	c.3140A>G	c.1624G>A	c.3139C>T	c.1132T>C	c.1633G>A	c.1624G>A	c.1633G>A	c.1357G>A
VAF (%)^a^	12	29	6	3	2	4	5	12	11	9.8	4.5	13
Amino Acid change	p.E542K	p.E545K	p.Q546H	p.E545K	p.H1047R	p.E542K	p.H1047Y	p.C378R	p.E545K	p.E542K	p.E545K	p.E453K
COSMIC^b^ Genomic mutation ID	55873227	55873239	55883555	55873239	55873195	55873227	55876499	55882697	55873239	55873227	55873239	55874585
Adverse events related to the venous malformation	DVT^c^ PE^d^ LIC^e^	LIC Chronic bleedings	DVT PE LIC	LIC Chronic bleedings	DVT PE LIC Chronic bleedings	DVT PE LIC Chronic bleedings	LIC DVT	LIC DVT	DVT PE LIC Chronic bleedings	LIC DVT	DVT PE LIC	DVT PE LIC Chronic bleedings
Alpelisib (mg per day)	250	250	250	250	50	250	250	250	250	250	50	250
Volume of the malformation (cm^3^)												
Prior to alpelisib introduction	9490800	2250120	587618	1487490	2468150	1319740	6683090	907853	1256600	2695840	615579	2081620
Six months following alpelisib introduction	5429780	530136	470094	797895	1913930	1198400	1705040	707840	663208	1929580	517086	1177580
Volume change following alpelisib (%)	−42.7	−76.4	−20	−46.3	−22.4	−9.2	−74.5	−22	−47.2	−28.4	−16	−43.3
Treatments prior to alpelisib	Scleroth.^f^ Surgeries Rapamycin	Scleroth. Surgeries Rapamycin	Scleroth. Surgeries Rapamycin	Rapamycin	Scleroth. Rapamycin	Scleroth. Surgeries	Surgeries Rapamycin	Scleroth. Surgeries Rapamycin	Surgeries Rapamycin	Scleroth. Surgeries Rapamycin	Scleroth. Surgeries Rapamycin	Scleroth. Rapamycin

	Patient 13	Patient 14	Patient 15	Patient 16
Age (years)	24	52	5	2
DNA changes	c.3139C>T	c.1258T>C	c.1633G>A	c.1624G>A
VAF (%)	10	2	6	2.2
Amino Acid change	p.H1047Y	p.C420R	p.E545K	p.E542K
COSMIC Genomic mutation ID	55876499	55874020	55873239	55873227
Adverse events related to the venous malformation	LIC Chronic bleedings	LIC DVT	DVT PE LIC	LIC DVT
Alpelisib (mg per day)	250	250	50	50
Volume of the malformation (cm^3^)				
Prior to alpelisib introduction	3321126	790047	58892	279460
Six months following alpelisib introduction	3013445	703439	28032	240847
Volume change following alpelisib (%)	−9.2	−10.9	−52.4	−13.8
Treatments prior to alpelisib	Scleroth. Surgeries Rapamycin	Scleroth. Surgeries Rapamycin	Surgeries Rapamycin	Rapamycin

^a^*VAF* Variant allele frequency, corresponds to the percentage of alternate or mutant reads to total reads detected by next generation sequencing

^b^*COSMIC* Catalogue Of Somatic Mutations In Cancer

^c^*DVT* Deep venous thrombosis

^d^*PE* Pulmonary embolism

^e^*LIC* Localized intravascular coagulation

^f^*Scleroth.* Sclerotherapies

**Table 2:** Patient characteristics with *TEK*-related venous malformations

	Patient 17	Patient 18	Patient 19	Patient 20	Patient 21	Patient 22	Patient 23	Patient 24	Patient 25
Age (years)	16	36	11	22	29	28	52	37	49
DNA changes	c.2742A>C	c.2742A>C	c.2742A>C	c.2742A>C	c.2742A>C	c.2742A>C	c.2742A>C	c.2742A>C	c.2742A>C
VAF (%)^a^	7	4	3	2	3	10	5	17.4	1.7
Amino Acid change	p.L914F	p.L914F	p.L914F	p.L914F	p.L914F	p.L914F	p.L914F	p.L914F	p.L914F
COSMIC^b^ Genomic mutation ID	59134128	59134128	59134128	59134128	59134128	59134128	59134128	59134128	59134128
Adverse events related to the venous malformation	LIC^c^ Chronic bleedings	LIC Chronic bleedings	DVT^d^ PE^e^ LIC	DVT PE LIC	LIC DVT	LIC DVT	LIC Chronic bleedings	LIC DVT	DVT PE LIC
Alpelisib (mg per day)	50	250	50	250	250	250	250	250	250
Volume of the malformation (cm^3^)									
Prior to alpelisib introduction	1447195	3453560	1424180	489565	478704	998287	1060207	5651740	14818100
Six months following alpelisib introduction	1332726	2944040	1331550	289778	433612	657466	481073,8	3875720	6890640
Volume change following alpelisib (%)	−7.9	−14.7	−6.5	−40.8	−9.4	−34.4	−54.6	−31.4	−53.5
Treatments prior to alpelisib	Scleroth.^f^ Surgeries Rapamycin	Scleroth. Surgeries Rapamycin	Scleroth. Surgeries Rapamycin	Scleroth. Surgeries Rapamycin	Scleroth. Surgeries Rapamycin	Scleroth. Surgeries Rapamycin	Scleroth. Surgeries Rapamycin	–	–

^a^*VAF* Variant allele frequency, corresponds to the percentage of alternate or mutant reads to total reads detected by next generation sequencing

^b^*COSMIC* Catalogue Of Somatic Mutations In Cancer

^c^*LIC* Localized intravascular coagulation

^d^*DVT* Deep venous thrombosis

^e^*PE* Pulmonary embolism

^f^*Scleroth.* Sclerotherapies

## Discussion

In this study, we successfully developed a mouse model of *PIK3CA*-related capillary venous malformations that accurately mimics the patient phenotype. We discovered that the malformations are partially driven by the AKT pathway. Alpelisib, which was tested in both mice and patients, showed significant efficacy in improving capillary venous malformations. The *PIK3CA*^*Tie2R-CreER*^ mouse model faithfully replicates the clinical, radiological, histological, and biological anomalies observed in individuals affected by venous malformations. Indeed, these malformations can manifest as isolated or diffused occurrences, appearing in various locations such as the head and neck region, trunk, and extremities as observed in the *PIK3CA*^*Tie2R-CreER*^ mouse model. In clinical terms, venous malformations are characterized by a soft, compressible mass presenting a bluish/purple superficial appearance, occasionally featuring palpable thrombi. They can be well-defined or diffuse and infiltrative akin to the *PIK3CA*^*Tie2R-CreER*^ mouse model. At the radiological level, MRI imaging is the mainstay of imaging of venous malformation and lesions identified in the *PIK3CA*^*Tie2R-CreER*^ mouse model are observed in patients. At the histological level, venous malformations are characterized by enlarged venous channels lined by a single flattened layer of endothelial cells surrounded by sparse, irregularly distributed smooth muscle cells. These lesions often demonstrate local hemophagocytosis and thrombosis, features observed in the *PIK3CA*^*Tie2R-CreER*^ mouse model. Biologically, vascular malformations are characterized by the presence of local or disseminated intravascular coagulation. Venous malformations are commonly linked to thrombosis and pulmonary embolism, as seen in our model, alongside instances of chronic bleeding. These bleedings and pulmonary embolisms stand as causes of death within this population. Notably, our mouse model exhibited signs of pulmonary embolism and traits indicative of chronic bleedings. These characteristics highlight the potential of the model to better understand the mechanism of disease progression and to serve as a new platform for drug testing.

The underlying cause of isolated or complex vascular malformations is primarily attributed to genetic activation of the PI3Kα/AKT pathway in endothelial cells. Tie2 is expressed in endothelial cells and has been observed in arteries.^27,28^ Since we induced the Cre recombinase in adult mice, these findings are in line with a recent report indicating that mutations in *PIK3CA* acquired postnatally in mice do not result in vascular anomalies.^16^ Gain-of-function mutations in *TEK* and *PIK3CA* are prevalent in the majority of venous malformations.^4^ Interestingly, TEK directly signals through PI3Kα, explaining the similarity of malformations and their responsiveness to alpelisib.^2^

Surprisingly, our findings using either genetic manipulations or pharmacological intervention indicate that AKT does not play a critical role in the development of the disease. Although we did not directly compare PIK3CA^AKT1AKT2KO^ mice and PIK3CA^Tie2-CreER^ mice treated with miransertib, we observed a severe outcome with uncontrolled disease progression in both sets of mice, accompanied by inhibited AKT phosphorylation in vascular malformations, indicating successful target engagement. This implies that PI3Kα likely signals through pathways different from AKT. However, recent reports of *AKT3* somatic mutations associated with a vascular phenotype.^29,30^ suggest a potential partial compensation for the loss of other isoforms. Additional studies will be necessary to provide further clarification.

Our newly created preclinical model is more severe compared to previous models that utilized human variants, thanks to the modified *PIK3CA* transgene that we are using. This model serves as a powerful tool for drug screening, enabling us to demonstrate the efficacy of alpelisib and its promising results in heavily pretreated patients. The remarkable aspect of this series is the notable clinical and radiological improvement seen in all these patients who were unresponsive to rapamycin treatment and underwent multiple surgical and radiological procedures. These findings validate the high sensitivity of vessels to PI3Kα inhibition.^18,31^

This work opens new therapeutic avenues for patients with *TEK*-related vascular malformations, a severe and debilitating condition that currently lacks approved treatment. Presently, patients are managed with laser, surgical, and radiological procedures, along with symptomatic treatments and anticoagulation. If confirmed in randomized controlled trials, such new targeted therapy could prove beneficial for patients with *TEK*-related vascular malformations, potentially becoming a cornerstone of the new therapeutic strategy for their care.

As a result of real-world evidence from the EPIK P1 clinical trial (NCT04285723), alpelisib has recently received accelerated approval from the US FDA for patients with PROS (age >2 years).^21^ Alpelisib is linked to adverse events such as hyperglycemia, alopecia, diarrhea, and vomiting. In the EPIK P1 clinical trial, adverse events were less severe and occurred less frequently, likely attributable to the lower dose utilized compared to oncology clinical trial.^32^ In the EPIK P1 clinical trial, the most common adverse events were grade 1 alopecia (16.7%), diarrhea (15.8%), hyperglycemia (12.3%), and aphthous ulcers (10.5%).^32^ It is worth noting that the doses of alpelisib that we have used here are based on the real-world data evidence where no pharmacokinetics analyses were available.^17,18,20,21,33^ The ongoing EPIK P2 clinical trial (NCT04589650) seeks to better define the appropriated dosages, to confirm the initial findings, and MRI follow-ups of these patients will help assess the impact of alpelisib on different tissues, especially venous malformations. Finally, the detection of altered levels of circulating metabolites upon starting alpelisib could lay the groundwork for future identification of biomarkers.

In conclusion, we have presented a novel mouse model of vascular malformations and provided encouraging preliminary evidence of alpelisib’s efficacy in patients with either *PIK3CA* or *TEK*-related capillary venous malformations. Further clinical studies are necessary to validate and corroborate our findings.

## Methods

### Animals

Here, we crossed homozygous R26StopFLP110*.^34^ (stock no. 012343, Jackson Laboratory) with *Tie2 Cre-ER* mice.^35^ (Infrafrontier). Following procedures previously described,^20^ we obtained R26StopFLP110*+/− × *Tie2 Cre-ER* + (henceforth *PIK3CA*^*Tie2-CreER*^) and R26StopFLP110*+/− × *Tie2 Cre-ER-* (henceforth *PIK3CA*^*WT*^) mice. We also generated *Tie2 Cre-ER+* mice with a homozygous R26StopFLP110* mutation (henceforth *PIK3CA*^*HO*^). To generate tissue-specific p110*-transgenic mice, a cloned loxP-flanked neoR-stop cassette was inserted into a modified version of pROSA26-1 followed by the p110* coding sequence, a frt-flanked IRES-EGFP cassette and a bovine polyadenylation sequence (R26StopFLP110*).^36^ To follow *Cre* recombination, *PIK3CA*^*WT*^ and *PIK3CA*^*Tie2-CreER*^ mice were interbred with *Gt(ROSA)26Sor*^*tm4(ACTB-tdTomato,-EGFP)Luo/J*^ mice.

Animals were housed at a constant ambient temperature in a 12-h light cycle and fed ad libitium with regular chow food (2018 Teklad global 18% protein rodent diets, 3.1 Kcal/g, Envigo). *Ministère de l’Enseignement Supérieur, de la Recherche et de l’Innovation* approved the animal procedures *(APAFIS N°20439-2018121913526398 and 2021110914486827)*. All appropriate procedures were followed to ensure animal welfare. For induction, all of the mice studied received at the age of 4 weeks a daily dose of 1 mg tamoxifen through oral gavage for 5 consecutive days. Before the sacrifice procedure, mice were harvested over a 12-h period.

*PIK3CA*^*Tie2-CreER*^ mice were interbred with *AKT1*^*flox/flox*^ obtained from The Jackson Laboratory (Stock #026474) to generate mice with *PIK3CA* gain-of-function mutation in venous endothelial cells but lacking *AKT1* (*PIK3CA*^*Tie2-CreER-AKT1KO*^ mice; henceforth *PIK3CA*^*AKT1KO*^). *PIK3CA*^*Tie2-CreER*^ mice were also interbred with *AKT2*^*-/-*^ mice obtained from The Jackson Laboratory (Stock# 006966) to generate mice with *PIK3CA* gain-of-function mutation in venous endothelial cells but lacking *AKT2* (*PIK3CA*^*Tie2-CreER-AKT2KO*^ mice; henceforth *PIK3CA*^*AKT2KO*^). Lastly, *PIK3CA*^*AKT1KO*^ and *PIK3CA*^*AKT2KO*^ were interbred to generate mice with *PIK3CA* gain-of-function mutation in venous cells lacking both *AKT* genes (*PIK3CA*^*Tie2-CreER-AKT1AKT2-KO*^ mice; henceforth *PIK3CA*^*AKT1AKT2-KO*^).

*PIK3CA*^*W*T^ and *PIK3CA*^*Tie2-CreER*^ mice were treated with the p110α inhibitor alpelisib [MedChem Express, Germany; 50 mg.kg^−1^ in 0.5% carboxymethylcellulose (Sigma Aldrich), daily p.o.] or a vehicle [0.5% carboxymethylcellulose (Sigma Aldrich), daily p.o.]. Treatment was started either 1 week (preventive study) or 4 weeks (therapeutic study) following Cre induction. *PIK3CA*^*W*T^ and *PIK3CA*^*Tie2-CreER*^ mice were also treated with either the Akt inhibitor miransertib and mTOR inhibitor rapamycin or a vehicle at a concentration of respectively 30 mg/kg and 4 mg/kg (MedChem Express, Germany) both diluted in a solution of 5% DMSO (Sigma Aldrich), 40% PEG-300 (Sigma Aldrich), 5% Tween (Euromedex) and 45% saline (Cooper). Both treatments were administered every day through oral gavage. The last dose of drug or vehicle was administered approximately 3 h before sacrifice. All mice were fasted overnight before blood glucose measurement (Accuchek Performa, Roche Diagnostic), MRI and sacrifice. Every solution was initially prepared over a span of 5–7 days and then systematically remade every 7 days thereafter.

### Magnetic resonance imaging (MRI) evaluation

All images were acquired with a 4.7-T small-animal MRI system (BioSpec USR47/40; Bruker BioSpin, Ettlingen, Germany) on the “Plateforme Imageries du Vivant, Université de Paris, PARCC, INSERM, Paris, France.” Mice underwent whole body magnetic resonance imaging (MRI) using 3D T2 weighted sequences with and without fat saturation. Whole body vascular malformations volumetric evaluation on MRI was performed with 3D Slicer software using manual segmentation tools.^37^

### Blood and plasma analyses

Mouse blood samples were collected in EDTA-coated tubes. To measure complete blood count, fresh blood samples were analyzed on a hematology analyzer (ProCyte Dx; IDEXX Laboratories). The remaining blood samples were centrifuged at 500 × *g* for 10 min to collect plasma.

### ELISA & multi-spot assay system (Meso Scale Diagnostics)

Mouse-specific D-dimers were measured in plasma mice samples using enzyme immunoassay kit from Cusabio Biotech (ref# CSB-E13584) following the manufacturer’s instructions and analyzed with a Tecan Infinite^®^ 200 PRO.

Levels of total and phosphorylated (Ser^473^) AKT protein in mice skin lysates was assessed using Meso Scale Diagnostics (MSD) multi-spot assay system (Phospho(Ser473)/Total Assay Whole Cell Lysate Kit ref K15100D) and read with Meso® QuickPlex SQ 120MM, Model 1300. Data were analyzed with MSD Discovery Workbench and calculated according to manufacturers’ instructions (%Phosphoprotein = ((2*Phospho-signal)/(Phospho-signal + Total signal)) * 100).

### Morphological analyses

Mouse tissues (all from abdominal skin) were fixed in 4% paraformaldehyde and paraffin-embedded. Tissue sections (4 µm thick) were stained with hematoxylin and eosin (H&E).

### Proliferation index evaluation

Proliferative cells were detected in abdominal skin tissue sections using KI67 immunostaining. Four-µm sections were incubated with a KI67 (Supplementary Table S1) primary antibody, and the appropriate Alexa-Fluor conjugated secondary antibody (Thermo Fisher Scientific). Image acquisition was performed with the Eclipse Ni-E(Nikon) at 400× magnification (numerical aperture of the objective: 0.75). Between 8 to 12 randomly selected fields (with the number depending on the size of the tissue) were taken for each section. Proliferation index was evaluated using QuPath(v0.4.2).^38^ as well as Cellpose extension.^39^ and a machine learning object classification method. Briefly, we created a sparse image containing all randomly-acquired images in QuPath software and then used the Cellpose pre-trained model cyto2 to identify all nuclei. Object classification using machine learning was performed and 3 classes were created: DAPI positive, KI67 positive and Other corresponding to potential artefacts.

Machine learning was chosen over the threshold method to overcome tissue staining heterogeneity that would not be as accurate. Consequently, this object classification was performed for each mouse. The ground truth of KI67 classification was determined and validated by the experts according to morphological criteria and the intensity. For each sparse image, we finally have a number of total DAPI-positive only nuclei, and KI67-positive nuclei. To calculate the ratio, we added the number of DAPI-positive and KI67-positive cells to obtain the total number of nuclei, subdivided by KI67-positive only cells. Nuclei classified as Other were excluded and subtracted from calculation.

### Immunohistochemistry and immunofluorescence

Paraffin-embedded mouse tissue sections were submitted to antigen retrieval protocols in either citrate or tris-EDTA buffer at high temperature (120 °C) and pressure with a pressure cooker. Four-µm sections were incubated with primary antibodies (Supplementary Table S1) overnight. For immunofluorescence, samples were then incubated with appropriate Alexa Fluor-conjugated secondary antibodies (Thermo Fisher Scientific) and analyzed using the Eclipse Ni-E (Nikon). Serial sectioning has been performed when antibodies were produced from the same species and GFP staining was used to localize the same area to acquire. Thirty-µm sections were submitted to the same protocol and were acquired using a Zeiss LSM 700 confocal microscope and Imaris Viewer 10.1.0 was used to create 3D images and ImageJ for the 2D images.

For immunohistochemistry, samples were then exposed to relevant horseradish peroxidase (HRP)-linked secondary antibodies and analyzed with the E800 microscope (Nikon).

### Western blot

Tissues were crushed and then lysed in RIPA lysis buffer supplemented with phosphatase and protease inhibitors. Protein concentrations were determined through the bicinchoninic acid method (Pierce). Protein extracts were analyzed by Western blot where the transfer membrane was incubated with a primary antibody (Supplementary Table S1) followed by the appropriate peroxidase-conjugated secondary antibody (dilution 1:10,000). Chemiluminescence were acquired using a Chemidoc MP and bands were quantitated with the Image Lab Software (Bio-Rad Laboratories).

### Tissue digestion

Skin of *PIK3CA*^*WT*^ and subcutaneous venous malformations of *PIK3CA*^*Tie2-CreER*^ mice were harvested and rinsed with PBS 1X (Gibco). For immunofluorescence and Western blot experiments, approximately 4 cm2 of abdominal skin was collected from the upper to the lower limbs at the time of sacrifice. For flow cytometry studies, almost all the skin from the front and back, spanning from the neck to the lower limbs, was collected to maximize the available material. After cutting them into small pieces, tissues were incubated in a digestion buffer added with DNAse (0.1 mg/mL), Dispase I (0.8 mg/mL) and Collagenase P (0.2 mg/mL) in 10 mL of RPMI (Gibco) and incubated for 40 min at 37 °C on a GentleMACS system (Miltenyi). Following dissociation, tissues were filtered (70 µm, Clearline), centrifuged 5 min at 250 × *g*, and resuspended in PBS.

### Flow cytometry

After digestion, cells were incubated with conjugated primary antibodies (Supplementary Table S1). Appropriated isotype controls were used.

To isolate bone marrow, we first dissected tibia and femur and then removed skeletal muscles, ligaments, and tendons. Cells were flushed from the marrow using 1-ml syringe containing phosphate-buffered saline (PBS) 1X. Cell suspension was centrifuged at 500 × *g* within 5 min and filtered (70 µm, Clearline). Cells were incubated with conjugated primary antibodies (Supplementary Table S1). Cells were then incubated with conjugated primary antibody (Supplementary Table S1). Appropriated isotype controls were used.

All samples were analyzed using the Sony ID7000 and Sony ID7000 software. Final analysis and quantification were conducted with the Kaluza software.

### Phosphoflow cytometry

Following 10 days of treatment with either the vehicle or alpelisib, subcutaneous skin of *PIK3CA*^*WT*^ mice and subcutaneous vascular malformations of *PIK3CA*^*Tie2-CreER*^ mice were harvested and digested. Cells were resuspended in 100 µL PBS in a 96-well round-bottomed plate (ThermoFisher). Cells were incubated with conjugated primary antibodies (Supplementary Table S1) for cell surface markers, rinsed with BD Pharmingen™ Stain Buffer FBS and permeabilized with BD™ Phosphoflow Perm buffer III for 30 min at 4 °C. Staining for intracytoplasmic markers (Supplementary Table S1) was performed for 30 min at 4 °C followed by cell fixation with BD Cytofix™. Appropriated isotype controls were used. Cells were analyzed using the Sony Spectral ID7000 apparatus and all flow data were processed and analyzed with the Sony ID7000 software.

### Imaging flow cytometry (Amnis ImageStream)

Skin from *PIK3CA*^*WT*^ and *PIK3CA*^*Tie2-CreER*^ mice were rinsed in phosphate buffered saline 1X, cut in small pieces and digested as detailed below. After dissociation, cell suspensions were filtered (70 µm, Clearline), centrifuged 10 min at 350 × *g*, and resuspended in phosphate buffered saline solution 1X supplemented with 2% fetal bovine serum and EDTA at 0.5 m/mol. After these procedures, cells were transferred in microtubes and stained for Tie2 conjugated antibody (Supplementary Table S1). All mice expressed the GFP and Tie2-Cre recombinase, with or without the transgene. Samples were run on the ImageStream ISX mkII (Amnis part of Luminex) apparatus that combines flow cytometry with detailed cell imaging. Magnification (40X) was used for all acquisitions. Data were extracted with the INSPIRE software (Amnis part of Luminex) and analyzed with the IDEAS software (v.6.2, Amnis part of Luminex).

### Targeted LC–MS metabolites analyses

Blood samples were obtained in EDTA tubes for plasma analysis and EDTA-free tubes for serum analysis. Plasma and serum were obtained after blood centrifugation at 500 × *g* for 10 min and immediately snap-frozen in liquid nitrogen. For the LC–MS analyses, metabolites were extracted as previously described.^20^ Briefly, the extraction solution, which was composed of 50% methanol, 30% ACN, and 20% water, was added to the plasma or serum volume at a ratio of 200 µl per 10 µl. Afterwards, samples were vortexed for 5 min at 4 °C and centrifuged at 16,000 × *g* for 15 min at 4 °C. The supernatants were collected and stored at −80 °C until the analyses were performed.

LC–MS analyses were conducted using a QExactive Plus Orbitrap mass spectrometer equipped with an Ion Max source and a HESI II probe coupled to a Dionex UltiMate 3000 UPLC system (Thermo). External mass calibration was performed using the standard calibration mixture every 7 d as recommended by the manufacturer. Five microliters of each sample were injected onto guard-column protected (Millipore) Zic‐pHilic columns for liquid chromatography separation. Buffer A was 20 mM ammonium carbonate and 0.1% ammonium hydroxide (pH 9.2) and buffer B acetonitrile. The chromatographic gradient was run at a flow rate of 0.200 μl/min as follows: linear gradient from 80 to 20% B for 0–20 min, linear gradient from 20 to 80% B for 20–20.5 min and held at 80% B for 20.5–28 min. The mass spectrometer was operated in full-scan polarity switching mode with the spray voltage set to 2.5 kV and the heated capillary held at 320 °C. The sheath gas flow was set to 20 units, the auxiliary gas flow to 5 units, and the sweep gas flow to 0 units. The metabolites were detected across a mass range of 75–1000 m/z at a resolution of 35,000 (at 200 m/z) with the AGC target set to 106 and maximum injection time to 250 ms. Lock masses were used to ensure mass accuracy below 5 ppm.

Data were acquired with the Thermo Xcalibur 4.0.27.13 software (Thermo). The peak areas of metabolites were determined using the Thermo TraceFinder 3.3 SP1 software (Thermo) and identified by the exact mass of each singly charged ion and by known retention time in the HPLC column.

### Patients

This study was conducted on 25 patients, including 7 children who were followed at *Hôpital Necker Enfants Malades* (Paris, France).^17^ and 2 adults who were followed at the *L’Hôtel-Dieu de Québec* du CHU de Québec – Université Laval (Québec, Canada). The patients followed at Necker were under a protocol that was approved by the Agence Nationale de Sécurité du Médicament et des Produits de Santé (ANSM). In this study, written informed consent was obtained from all adult patients and from the parents of all pediatric patients. Alpelisib, which was compassionately offered by Novartis, was given every morning during breakfast at an oral dose of 250 mg/day to adults and 50 mg/day to pediatric patients.^17^

Adverse events were graded according to Common Terminology Criteria for Adverse Events [CTCAE] system (version 4.03) and coded by preferred term using the Medical Dictionary for Regulatory Activities [MedDRA] system (version 24.0). Patients underwent magnetic resonance imaging (MRI) prior to alpelisib introduction and this exam was repeated after 6 months of more of treatment. Patients underwent localized MRI using T2 weighted sequences with fat suppression including fat saturation (FAT-SAT), short TI inversion recovery (STIR) and DIXON sequences. Baselines acquisitions were registered with the follow-up MRI and then cropped to obtain a similar field of view on which segmentation of venous malformation was performed with 3D Slicer as previously described for the MRI in mice. To confirm the venous malformations, all patients underwent Doppler ultrasound prior to drug initiation.

In human, skin biopsies were obtained from patients after informed consent. Control skin was obtained from surgical tissues removed for other reasons. Histology was systematically verified by a pathologist. Paraffin-embedded skin tissue sections were submitted to antigen retrieval protocols in either citrate or tris-EDTA buffer at high temperature (120 °C) and pressure with a pressure cooker. Four-µm sections were incubated with primary antibodies (Supplementary Table S1) overnight. Samples were then incubated with appropriate Alexa Fluor-conjugated secondary antibodies (Thermo Fisher Scientific) and analyzed using the Eclipse Ni-E (Nikon). P-S6RP and P-AKT^Thr308^ staining were quantified using the Ilastik v1.3.3post3 machine learning pixel classification opensource software. Afterwards, mean signal intensity and areas were measured with the Fiji v2.3.0/1.53f51 open-source image processing software (Quantity = Mean intensity × Area) and normalized by tissue area.^40^

KI67 staining was performed on human paraffin-embedded skin biopsie sections by immunohistochemistry. Proliferation index was evaluated using QuPath(v0.4.2).^38^ and Stardist extension.^41^ Then, object classification using machine learning was performed and 3 classes were created: Hematoxylin-positive, DAB-positive corresponding to Ki67-positive nuclei and Artefacts.^42,43^ To calculate the ratio, we added the number of hematoxylin-positive and DAB-positive cells to obtain the total number of nuclei, subdivided by DAB-positive only cells. Nuclei classified as Artefacts were excluded and subtracted from calculation.

### Data analysis and statistics

Data were expressed as means ± SEM. Survival curves were analyzed with the Mantel–Cox (log-rank) test. Differences between experimental groups were evaluated using analysis of variance (ANOVA), followed by the Tukey–Kramer post hoc test for statistically significant differences (*P* < 0.05). When only two groups were compared, Mann–Whitney tests were used. The statistical analysis was performed using GraphPad Prism software (version 10.0.0).

### Supplementary information

Supplementary Figures and Tables

Uncropped WB

Supplementary Data_Supp Fig 6

Supplementary Data_Supp Fig 12 and 13

Supplementary Data_Fig 17

## Data Availability

All data needed to evaluate the conclusions in the paper are present in the paper, in the Supplementary Materials or online for the LCM MS raw data.
