# Supplementary material for: Targeted therapy for capillary-venous malformations
Source: Signal Transduct Target Ther. 2024 Jun 17;9:146. doi: 10.1038/s41392-024-01862-9 (PMC11180659; doi:10.1038/s41392-024-01862-9)
Supplement: Supplementary file 1 — Supplementary Figures and Tables [file 41392_2024_1862_MOESM1_ESM.docx]

Supplementary Materials for

***Targeted therapy for capillary-venous malformations***

Lola Zerbib^1,2^, Sophia Ladraa^1,2^, Antoine Fraissenon^2,3,4,5^, Charles Bayard^1,2^, Marina Firpion^1,2^, Quitterie Venot^1,2^, Sanela Protic^1,2^, Clément Hoguin^1,2^, Amandine Thomas^2^, Sylvie Fraitag^6^, Jean-Paul Duong^1,6^, Sophie Kaltenbach^7^, Estelle Balducci^1,7^, Coline Lefevre^7^, Patrick Villarese^7^, Vahid Asnafi^1,2,7^, Christine Broissand^8^, Nicolas Goudin^9^, Ivan Nemazanyy^10^, Gwennhael Autret^11^, Bertrand Tavitian^11^, Christophe Legendre^1,2,12^, Nadia Arzouk^13^, Veronique Minard-Colin^14^, Caroline Chopinet^15^, Michael Dussiot^16^, Denise M. Adams^17^, Tristan Mirault^1,18^, Laurent Guibaud^2,3^, Paul Isenring^19^ and Guillaume Canaud^1,2,20,21†^.

Correspondence to: [guillaume.canaud@inserm.fr](mailto:guillaume.canaud@inserm.fr)

**This PDF file includes:**

Supplementary Figures. S1 to S17

Tables S1 to S2

**Supplementary Figure 1: Characterization of the *PIK3CA*-realted capillary venous malformation mouse model.** (**a**) Female and male body weights of *PIK3CA^WT^*and *PIK3CA^Tie2-CreER^* mice (n= 10 per group) following *Cre* recombination. (**b**) Coronal whole-body T2-weighted (T2) T2-weighted fat saturated (T2FS) magnetic resonance images (MRI) of *PIK3CA^WT^*and *PIK3CA^Tie2-CreER^* mice 5 weeks after *Cre* recombination with volumetric segmentation superimposed in 2D (T2FS 2D) and 3D (T2FS 3D). (**c**) Representative photographs of *PIK3CA^WT^*and *PIK3CA^Tie2-CreER^* mice 4 weeks after *Cre* recombination during sacrifice. (**d**) Representative Hematoxylin and eosin (H&E) staining of hemophagocytosis on the left panel (examples showed with arrows) and hemosiderin on the right panel (examples showed with arrows) in vascular malformations of *PIK3CA^Tie2-CreER^* mice. Scale bar: 10μm. (**e**) Representative H&E staining of pulmonary embolism (arrow) in *PIK3CA^Tie2-CreER^* mice. Scale bar: 10μm. (**f**) Clots (arrows) observed in vascular malformation in a *PIK3CA^Tie2-CreER^* mouse. Scale bar: 10μm. (**g**) Representative immunostaining of Tie2, α-smooth muscle actin (αSMA), podoplanin and leptin in the skin of *PIK3CA^WT^*and *PIK3CA^Tie2-CreER^* mice but also in the skin of healthy controls and patients with *PIK3CA*-related venous malformations. Scale bar: 10μm.

**Supplementary Figure 2: *Cre* recombinase is expressed in endothelial cells.** (**a**) GFP immunostaining in various organs of *PIK3CA^WT^*and *PIK3CA^Tie2-CreER^* mice. (**b**) Western blot of p110 and p110* in the skin of *PIK3CA^WT^*and *PIK3CA^Tie2-CreER^* mice (n= 3 per group). (**c**) Representative CD3 immunostaining in the skin of *PIK3CA^WT^*and *PIK3CA^Tie2-CreER^* mice and quantification (n= 3 mice per group). Scale bar: 10μm. (**d**) Representative coimmunofluorescence staining of CD163 and F4/80 in the skin of *PIK3CA^WT^*and *PIK3CA^Tie2-CreER^* mice and (**e**) quantification (n= 3 mice per group). Scale bar: 10μm. (**f**) Representative coimmunofluorescence staining of F4/80 and GFP in the skin of *PIK3CA^WT^*and *PIK3CA^Tie2-CreER^* mice. Scale bar: 10μm. (**g**) Flow cytometry experiments showing the percentage of GFP+CD45+ cells and (**h**) GFP+CD34+ cells isolated from the bone marrow of *PIK3CA^WT^* (n= 4 mice per group).

**Supplementary Figure 3: Vessel disorganization in the skin of PIK3CA^Tie2-CreER^ mice*.*** (**a**) Representative coimmunofluorescence staining of GFP and COUP-TFII in the skin of *PIK3CA^WT^*and *PIK3CA^Tie2-CreER^* mice. Scale bar: 10μm. A: artery and V: vein. (**b**) 3D and 2D (maximum intensity projection) representation of GFP and COUP-TFII in the skin of *PIK3CA^WT^*and *PIK3CA^Tie2-CreER^* mice on 30μm sections. Scale bar: 20μm.

**Supplementary Figure 4: Vessel disorganization in the skin of PIK3CA^Tie2-CreER^ mice*.*** (**a**) 3D and 2D (maximum intensity projection) representation of GFP and CD31 in the skin of *PIK3CA^WT^*and *PIK3CA^Tie2-CreER^* mice on 30μm sections. Scale bar: 20μm. E: erythrocytes. (**b**) 3D and 2D (maximum intensity projection) representation of GFP and Podoplanin in the skin of *PIK3CA^WT^*and *PIK3CA^Tie2-CreER^* mice on 30μm sections. Scale bar: 20μm. (**c**) 3D and 2D (maximum intensity projection) representation of GFP and Ephrin B2 in the skin of *PIK3CA^WT^*and *PIK3CA^Tie2-CreER^* mice. Scale bar: 20μm. (**d**) 3D and 2D (maximum intensity projection) representation of GFP and Endomucin in the skin of *PIK3CA^WT^*and *PIK3CA^Tie2-CreER^* mice on 30μm sections. Scale bar: 20μm.

**Supplementary Figure 5: *PIK3CA^Tie2-CreER^* mice demonstrate increased proliferation rate.** (**a**) Representative coimmunofluorescence staining of KI67 and GFP in the skin of *PIK3CA^WT^* and *PIK3CA^Tie2-CreER^* mice. Scale bar: 10 μm. (**b**) Representative immunofluorescence staining on serial sections of ERG, KI67 and GFP in the skin of *PIK3CA^WT^* and *PIK3CA^Tie2-CreER^* mice. Scale bar: 10 μm. (**c**) Representative images of the surface of GFP positive cells isolated from *PIK3CA^WT^* and *PIK3CA^Tie2-CreER^* mice using Amnis ImageStream.

**Supplementary Figure 6: Plasma metabolic changes observed in *PIK3CAWT* and *PIK3CA^Tie2-CreER^*.** Graphic example of metabolite modifications observed. AU: Arbitrary units.

**Supplementary Figure 7: *PIK3CA* allele dose effect in endothelial veins.** (**a**) Representative photography of *PIK3CA^WT^* and *PIK3CA^Tie2-HO^* mice 4 weeks following *Cre* recombination. (**b**) Female and male body weights of *PIK3CA^WT^*and *PIK3CA^Tie2-CreER^* mice (n= 10 per group) following *Cre* recombination. (**c**) Kaplan–Meier survival curves of *PIK3CA^WT^*, *PIK3CA^Tie2-CreER^* and *PIK3CA^Tie2-HO^* mice (n= 16 per group). (**d**) Coronal whole-body T2-weighted (T2) T2-weighted fat saturated (T2FS) magnetic resonance images (MRI) of *PIK3CA^WT^*, *PIK3CA^Tie2-CreER^* and *PIK3CA^Tie2-HO^* mice with volumetric segmentation superimposed in 2D (T2FS 2D) and 3D (T2FS 3D) (n= 3 mice per group) 5 weeks after tamoxifen administration. (**e**) Representative hematoxylin and eosin (H&E) of the skin of *PIK3CA^WT^*, *PIK3CA^Tie2-CreER^* and *PIK3CA^Tie2-HO^* mice. Scale bar: 10μm. (**f**) Representative H&E staining of multiple pulmonary embolisms (arrows) in *PIK3CA^Tie2-HO^* mice. Scale bar: 10μm. (**g**) Representative P-AKT^Thr308^ and P-S6RP immunostaining in the skin of *PIK3CA^WT^*and *PIK3CA^Tie2-CreER^* and *PIK3CA^Tie2-HO^* mice. Scale bar: 10μm. (**h**) P-AKT^Ser473^ Elisa quantification in the skin isolated from *PIK3CA^WT^*and *PIK3CA^Tie2-HO^* mice (n= 8 per group). (**i**) Complete blood count in *PIK3CA^WT^*, *PIK3CA^Tie2-CreER^* and *PIK3CA^Tie2-HO^* mice (n= 4-6 mice per group).

**Supplementary Figure 8: Phenotypic description of *PIK3CA^Tie2-CreER^*** **deleted for *AKT1*, *AKT2* or both isoforms.** (**a**) Representative photographs of *PIK3CA^WT^*, *PIK3CA^Tie2-CreER^*, *PIK3CA^AKT1KO^*, *PIK3CA^AKT2KO^* and *PIK3CA^AKT1AKT2-KO^* mice 6 weeks after *Cre* recombination. (**b**) Coronal whole-body T2-weighted (T2) T2-weighted fat saturated (T2FS) magnetic resonance images (MRI) of *PIK3CA^WT^*, *PIK3CA^Tie2-CreER^*, *PIK3CA^AKT1KO^*, *PIK3CA^AKT2KO^* and *PIK3CA^AKT1AKT2-KO^* mice 6 weeks after *Cre* recombination with volumetric segmentation superimposed in 2D (T2FS 2D) and 3D (T2FS 3D). (**c**) Complete blood count in *PIK3CA^WT^*, *PIK3CA^Tie2-CreER^*, *PIK3CA^AKT1KO^*, *PIK3CA^AKT2KO^* and *PIK3CA^AKT1AKT2-KO^* mice (n= 6-9 per group).

**Supplementary Figure 9: Phenotypic description of *PIK3CA^WT^* and *PIK3CA^Tie2-CreER^*** **treated with different drugs.** (**a**) Representative photographs of *PIK3CA^WT^* and *PIK3CA^Tie2-CreER^* mice 4 weeks after *Cre* recombination treated with either vehicle, rapamycin, miransertib or alpelisib. (**b**) Coronal whole-body T2-weighted (T2) T2-weighted fat saturated (T2FS) magnetic resonance images (MRI) of *PIK3CA^WT^* and *PIK3CA^Tie2-CreER^* mice 6 weeks after *Cre* recombination treated with either vehicle, rapamycin, miransertib or alpelisib with volumetric segmentation superimposed in 2D (T2FS 2D) and 3D (T2FS 3D).

**Supplementary Figure 10: Phenotypic description of *PIK3CA^WT^* and *PIK3CA^Tie2-CreER^*** **treated with either preventive or therapeutic alpelisib.** (**a**) Male body weights of *PIK3CA^WT^*and *PIK3CA^Tie2-CreER^* mice treated with either vehicle or preventive alpelisib (n= 10 per group) following *Cre* recombination. (**b**) Representative photographs of *PIK3CA^WT^* and *PIK3CA^Tie2-CreER^* mice 4 weeks after *Cre* recombination treated with either vehicle or preventive alpelisib. (**c**) Coronal whole-body T2-weighted (T2) T2-weighted fat saturated (T2FS) magnetic resonance images (MRI) of *PIK3CA^WT^* and *PIK3CA^Tie2-CreER^* mice 6 weeks after *Cre* recombination treated either vehicle or preventive alpelisib with volumetric segmentation superimposed in 2D (T2FS 2D) and 3D (T2FS 3D). (**d**) Adipose tissue volume measurement in the different group of mice base on MRI. (**e**) Flow cytometry experiments showing the percentage of GFP+CD31+ cells isolated from the skin of *PIK3CA^WT^* and *PIK3CA^Tie2-CreER^* mice treated either with vehicle or therapeutic alpelisib expressing P-AKT^Ser473^ (n= 5-7 mice per group).

**Supplementary Figure 11: Histological description of *PIK3CA^WT^* and *PIK3CA^Tie2-CreER^*** **treated with either preventive or therapeutic alpelisib.** (**a**) Representative coimmunofluorescence staining of aSMA and CD31 in the skin of *PIK3CA^WT^*and *PIK3CA^Tie2-CreER^* mice treated with either vehicle or preventive alpelisib. Scale bar: 10 μm. (**b**) Body weight evolution of *PIK3CA^Tie2-CreER^* mice treated from week 8 to 23 (n= 4 mice, mean±SEM).

**Supplementary Figure 12: Plasma metabolic changes observed in *PIK3CAWT* and *PIK3CA^Tie2-CreER^* treated with alpelisib.** Graphic example of metabolite modifications observed. AU: Arbitrary units.

**Supplementary Figure 13 (next): Plasma metabolic changes observed in *PIK3CAWT* and *PIK3CA^Tie2-CreER^* treated with alpelisib.** Graphic example of metabolite modifications observed. AU: Arbitrary units.

**Supplementary Figure 14: Proliferation rate in vascular malformations.** Representative KI67 immunostaining in skin biopsies of two controls and two patients and quantification. Scale bar: 250 μm.

**Supplementary Figure 15: Alpelisib improves patients with *PIK3CA* or *TEK*-related capillary venous malformations.** Representative photographs of the morphological changes observed in patients with either *PIK3CA*- or *TEK*-related capillary venous malformations receiving alpelisib for 6 months.

**Supplementary Figure 16: Radiological improvement of venous malformations in patients with *PIK3CA* or *TEK* gain-of-function mutations following alpelisib introduction.** T2-weighted fat saturated MRI sequence of patient 1,3, 5 and 17 before and after alpelisib introduction. In red, segmentation in 2D (left panel) and 3D (right panel).

**Supplementary Figure 17: Plasma metabolic changes observed ~~in~~ following alpelisib introduction in patients with either *PIK3CA-* or *TEK*-related capillary venous malformations.** Graphic example of metabolite modifications observed. AU: Arbitrary units.

**Supplementary Table 1: Antibodies used in the study**

| **Antibody target** | **Catalog** | | **Supplier** | | **Working dilution** | **Application** | | |  |
| --- | --- | --- | --- | --- | --- | --- | --- | --- | --- |
| p110α | 4249 | | Cell Signaling Technology | | 1:1000 | WB | | |  |
| p-AKT^Ser473^ | 4060 | | Cell Signaling Technology | | 1:1000 |  |  |  |  |
| α-Tubulin | t5168 | | Sigma | | 1:10000 |  |  |  |  |
| β-actine | 3700 | | Cell Signaling Technology | | 1:5000 |  |  |  |  |
| AKT1 | 75692 | | Cell Signaling Technology | | 1:1000 |  |  |  |  |
| AKT2 | 3063 | | Cell Signaling Technology | | 1:1000 |  |  |  |  |
| AKT | 9272 | | Cell Signaling Technology | | 1:1000 |  |  |  |  |
| p-S6RP | 5364 | | Cell Signaling Technology | | 1:1000/1:100 | WB/IF | | |  |
| GFP | ab13970 | | Abcam | | 1:1000/1:100 |  |  |  |  |
| p-ERK (p-p44) | 4376 | | Cell Signaling Technology | | 1:100 | IF | | |  |
| F4/80 | MCA497R | | AdB Serotec | | 1:100 |  |  |  |  |
| CD3 | ab16669 | | Abcam | | 1:100 |  |  |  |  |
| CD163 | ab182422 | | Abcam | | 1:200 |  |  |  |  |
| Perilipin-1 | 9349 | | Cell Signaling Technology | | 1:100 |  |  |  |  |
| α-SMA | a5228 | | Sigma | | 1:10000 |  |  |  |  |
| Ki67 | 12202 | | Cell Signaling Technology | | 1:100 |  |  |  |  |
| p-AKT^Thr308^ | 2965 | | Cell Signaling Technology | | 1:100 |  |  |  |  |
| Tie2 | AF762 | | R&D Biotechne | | 1:50 |  |  |  |  |
| Podoplanin | AF3244 | | R&D Biotechne | | 1:100 |  |  |  |  |
| COUPTFII | 6434 | | Cell Signaling Technology | | 1:200 |  |  |  |  |
| EphrinB2 | MA5-32740 | | ThermoFisher Scientific | | 1:100 |  |  |  |  |
| Endomucin | 14-5851-82 | | Invitrogen | | 1:500 |  |  |  |  |
| ERG | 97249 | | Cell Signaling Technology | | 1:500 |  |  |  |  |
| CD31 | PA516301 | | ThermoFisher Scientific | | 1:100 |  |  |  |  |
| Tie2-APC | 17-5987-82 | | Invitrogen | | 1:10 | FC | | |  |
| CD31-BV510 | 563089 | | BD Biosciences | | 1:10 |  |  |  |  |
| p-AKT^Ser473^-AF532 | 26580 | | Cell Signaling Technology | | 1:10 |  |  |  |  |
| GFP beads | A10514 | | Invitrogen | | 1 drop |  |  |  |  |
| Compensation beads | 01-2222-42 | | Invitrogen | | 1 drop |  |  |  |  |
| CD3-Pacific Blue | 1101070 | | Sony | | 1:10 |  |  |  |  |
| CD163-PE/Dazzle 594 | 1376580 | | Sony | | 1:10 |  |  |  |  |
| F4/80-APC | 1215580 | | Sony | | 1:10 |  |  |  |  |
| CD45-PerCP | 1115650 | | Sony | | 1:10 |  |  |  |  |
| CD11b-BV421 | 1106180 | | Sony | | 1:10 |  |  |  |  |
| CD206-AF700 | C068C2 | | Sony | | 1:10 |  |  |  |  |
| Podoplanin-APC | 127409 | | Biolegend | | 1:10 |  |  |  |  |
| Ki67-AF700 | 56-5698-82 | | Invitrogen | | 1:10 |  |  |  |  |
| CD31-BV421 | 1112120 | | Sony | | 1:10 |  |  |  |  |
|  | |  | |  | | |  |  | |
|  | |  | |  | | |  |  | |

WB: Western blot; IF: Immunofluorescence; FC: Flow cytometry

**Supplementary Table 2: Materials and buffers used in the study**

| **Name** | **Catalog** | **Supplier** | **Working dilution** |
| --- | --- | --- | --- |
| PBS 10X | ET330-A | Euromedex | 1X |
| RPMI | 61870 | Gibco |  |
| TBS 10X | ET220-B | Euromedex | 1X |
| TG-SDS 10X | EU510 | Euromedex | 1X |
| Citrate Buffer 10X | CBB999 | Cliniscience | 1X |
| 10X Tris-EDTA Buffer | K043 | Cliniscience | 1X |
| Tween20 | 2001-C | Euromedex | 0.5% |
| PEG400 | 8074851000 | Sigma-Aldrich |  |
| Pierce BCA protein assay kit | 23225 | Thermofischer |  |
| DNAse I | 10104159001 | Sigma-Aldrich | 0.1mg/mL |
| Dispase I | 4942086001 | Sigma-Aldrich | 0,8mg/mL |
| Collagenase P | 11213857001 | Sigma-Aldrich | 0,2mg/mL |
| 70µm cell-strainer | 141379C | Clearline |  |
| 96-wells round-bottomed plate | 163320 | Thermofischer |  |
| BD Pharmingen™ Stain Buffer FBS | 554656 | BD Pharmingen™ |  |
| BD™ Phosphoflow Perm buffer III | 558050 | BD Pharmingen™ |  |
| BD Cytofix™ | 554655 | BD Pharmingen™ |  |
| BD Cytofix/cytoperm™ | 512091KZ | BD Pharmingen™ |  |
| BD™ Perm/wash | 512091KZ | BD Pharmingen™ | 1X |
| Fluoromount aqueous mounting medium | F4680 | Sigma-Aldrich |  |
| Tamoxifen free base | T5648 | Sigma-Aldrich |  |
| Carboxymethylcellulose |  | Sigma-Aldrich |  |
| BYL-719 (Alpelisib) | HY-15244 | MedChem Express |  |
| Rapamycin | HY-10219 | MedChem Express |  |
| Miransertib | HY-19719 | MedChem Express |  |
| FBS | F7514 | Sigma-Aldrich |  |
| Paraformaldehyde 32% | 15714S | Euromedex | 4% |
| DAB | K3468 | DAKO |  |
| RIPA Lysis Buffer 10X | 20188 | EMD Millipore | 1X |
| BSA | 103570C | Euromedex | 0,03 |
| Fixation/Perm diluent | 00-5223-56 | Invitrogen |  |
| Fixation/Permeabilization concentrate | 00-5123-43 | Invitrogen | 0,25 |
| Permeabilization buffer 10X | 00-8333-56 | Invitrogen | 1X |
