# Supplementary material for: Targeted therapy for capillary-venous malformations
Source: Signal Transduct Target Ther. 2024 Jun 17;9:146. doi: 10.1038/s41392-024-01862-9 (PMC11180659; doi:10.1038/s41392-024-01862-9)
Supplement: Supplementary file 2 — Uncropped WB [file 41392_2024_1862_MOESM2_ESM.pptx]

## Slide 1
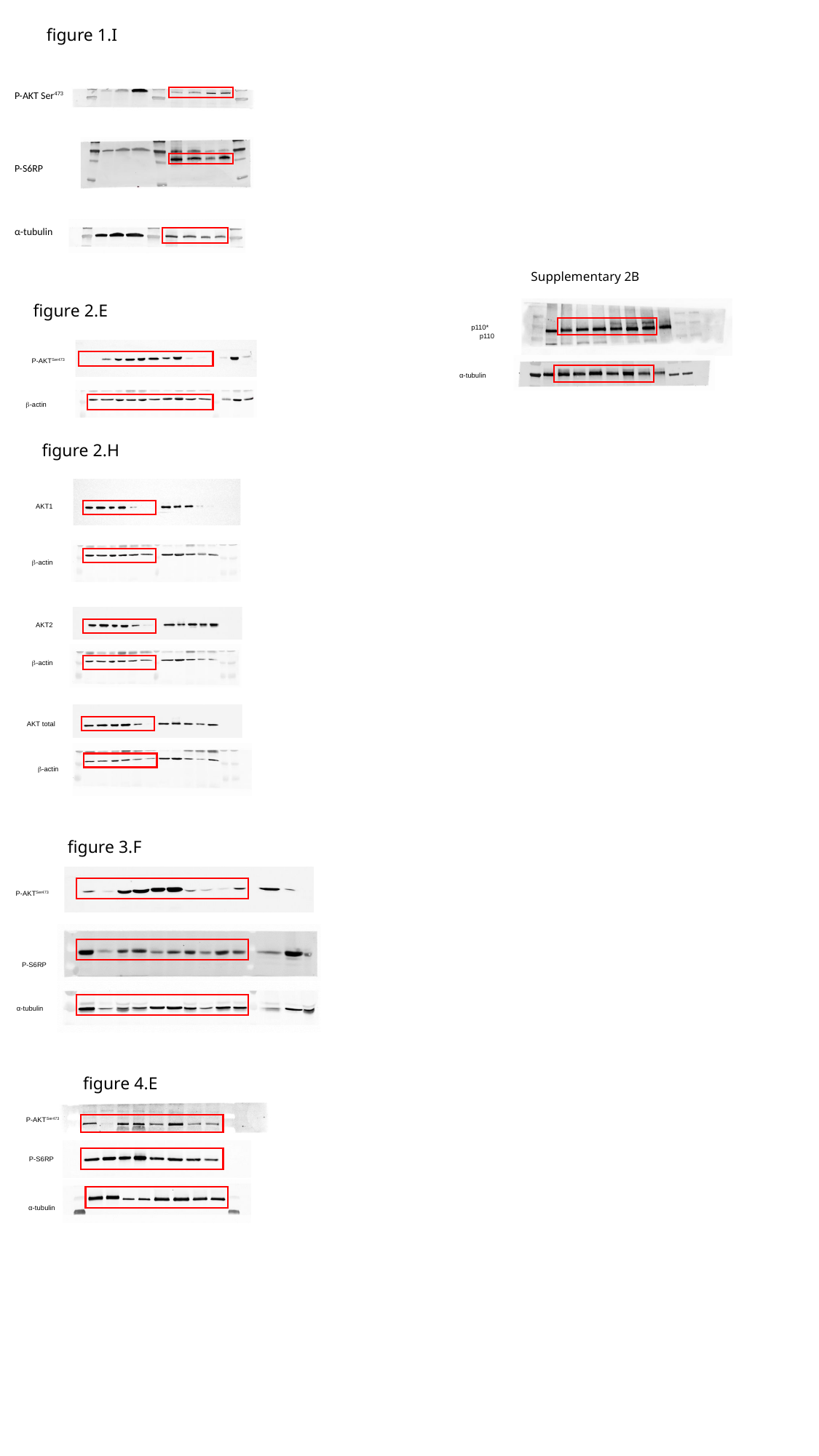

# figure 1.I
P-AKT Ser473
P-S6RP
α-tubulin
Supplementary 2B
figure 2.E
p110*
p110
P-AKTSer473
α-tubulin
b-actin
figure 2.H
AKT1
b-actin
AKT2
b-actin
AKT total
b-actin
figure 3.F
P-AKTSer473
P-S6RP
α-tubulin
figure 4.E
P-AKTSer473
P-S6RP
α-tubulin
